# Rational development of Stafib-2: a selective, nanomolar inhibitor of the transcription factor STAT5b

**DOI:** 10.1038/s41598-017-00920-3

**Published:** 2017-04-11

**Authors:** Nagarajan Elumalai, Angela Berg, Stefan Rubner, Linda Blechschmidt, Chen Song, Kalaiselvi Natarajan, Jörg Matysik, Thorsten Berg

**Affiliations:** 1 Institute of Organic Chemistry, Leipzig University, Johannisallee 29, 04103 Leipzig, Germany; 2 Institute of Analytical Chemistry, Leipzig University, Johannisallee 29, 04103 Leipzig, Germany; 3 Leids Instituut voor Chemisch Onderzoek, Universiteit Leiden, 2300 RA Leiden, The Netherlands

## Abstract

The transcription factor STAT5b is a target for tumour therapy. We recently reported catechol bisphosphate and derivatives such as Stafib-1 as the first selective inhibitors of the STAT5b SH2 domain. Here, we demonstrate STAT5b binding of catechol bisphosphate by solid-state nuclear magnetic resonance, and report on rational optimization of Stafib-1 (K_i_ = 44 nM) to Stafib-2 (K_i_ = 9 nM). The binding site of Stafib-2 was validated using combined isothermal titration calorimetry (ITC) and protein point mutant analysis, representing the first time that functional comparison of wild-type versus mutant protein by ITC has been used to characterize the binding site of a small-molecule ligand of a STAT protein with amino acid resolution. The prodrug Pomstafib-2 selectively inhibits tyrosine phosphorylation of STAT5b in human leukaemia cells and induces apoptosis in a STAT5-dependent manner. We propose Pomstafib-2, which currently represents the most active, selective inhibitor of STAT5b activation available, as a chemical tool for addressing the fundamental question of which roles the different STAT5 proteins play in various cell processes.

## Introduction

**Figure 1:**
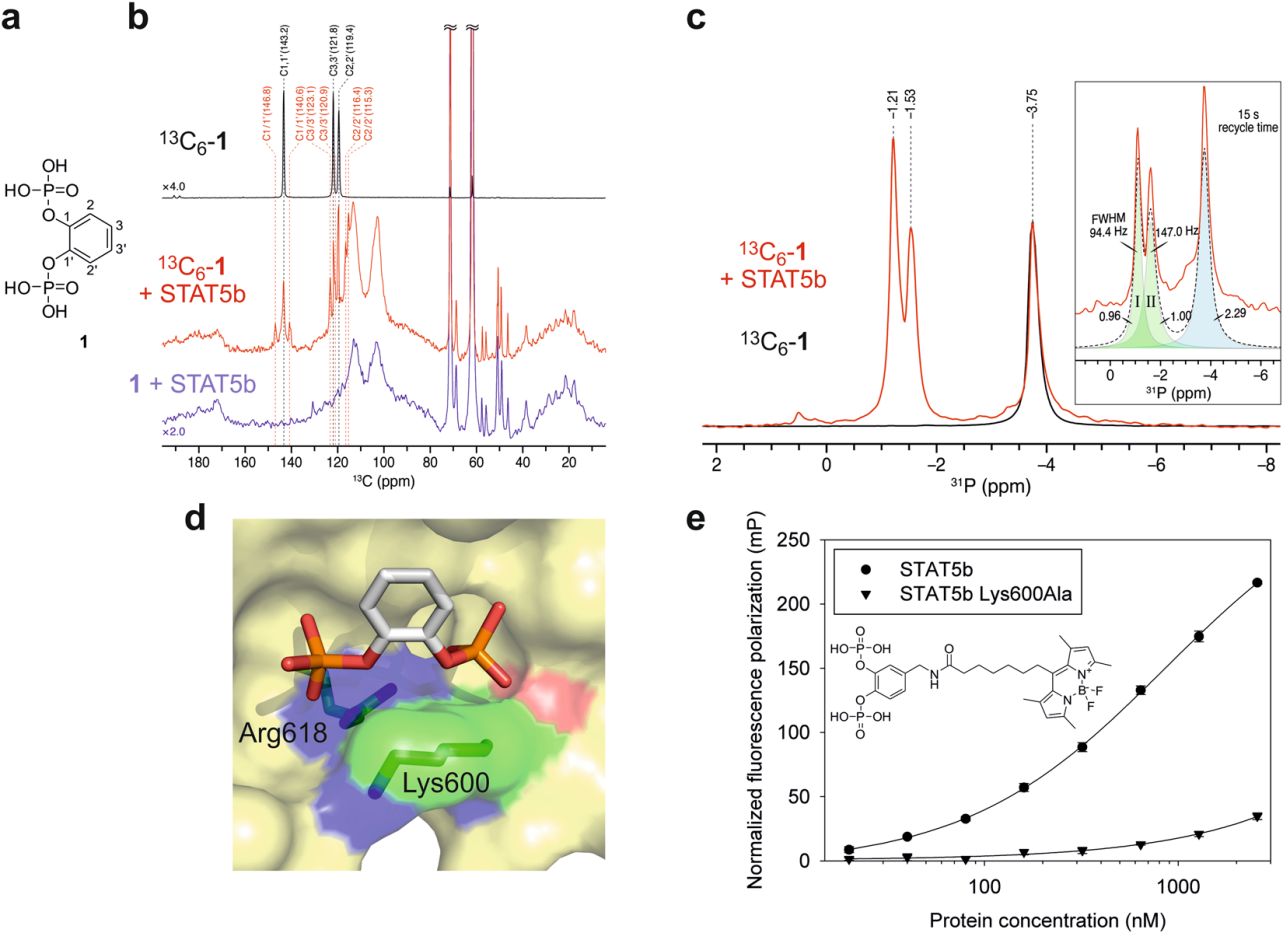
Binding of catechol bisphosphate (**1**) to the STAT5b SH2 domain. (**a**) Chemical structure of **1**. (**b**) ^13^C DP/MAS-NMR of ^13^C_6_-**1** in buffer in the absence (black) and presence (red) of STAT5b. The spectrum of non-isotopically enriched **1** in the presence of STAT5b is shown in blue. (**c**) ^31^P DP/MAS-NMR of ^13^C_6_-**1** in the absence (black) and presence (red) of STAT5b (recycle delay: 2.5 s) Inset: a pure Lorentzian function was applied to fit the experimental spectrum of ^13^C_6_-**1** in the presence of STAT5b (recycle delay: 15 s). Deconvolution produced two fits (sites I and II) of STAT5b-bound ^13^C_6_-**1** with the equal relative integral areas. The sum of the Lorentzian fits is shown as dotted curve. (**d**) Binding mode of **1** to the STAT5b SH2 domain as predicted by AutoDock Vina^15^. The figure was generated using PyMol^37^. (**e**) Binding between a fluorophore-labelled derivative of **1** to STAT5b wild-type (previously published in)^15^ or the STAT5b Lys600Ala mutant analysed by fluorescence polarization. Error bars represent standard deviations from three independent experiments, except for STAT5b Lys600Ala at 2.56 µM (n = 2).

Transcription factors orchestrate cellular signalling by regulating transcription of their target genes, thus allowing precise regulation of cellular phenotype^1^. They do not possess enzymatic activities, making their functional manipulation with cell-permeable small molecules more challenging. The transcription factors STAT5a and STAT5b in particular are highly homologous^2^ and are frequently referred to jointly as “STAT5”, implying that they carry out identical functions. However, while some protein functions are indeed redundant, others are not. For example, although both STAT5a and STAT5b are constitutively activated in numerous human cancers, including human leukaemias harbouring the Philadelphia chromosome^3^ which leads to expression of the Bcr-Abl fusion protein, the inhibition of STAT5b was shown to reduce tumour cell proliferation more than the inhibition of STAT5a did^4, 5^. Small-molecule inhibitors which differentiate between the two STAT5 proteins would be highly beneficial for clarifying their individual roles. The most effective and selective approach by which to inhibit STAT proteins involves functional inhibition of the protein-protein interaction domain, the Src homology 2 (SH2) domain^6, 7^. However, for most STAT5 inhibitors developed to date, including chromone-based compounds^8, 9^, fosfosal^10^, salicylic acid-based STAT5 inhibitors^11, 12^, an adenosine-5′-monophosphate derivative^13^, and an osmium complex^14^, selectivity for one STAT5 protein over the other was either minimal or not reported. We recently presented catechol bisphosphate (**1**, Fig. 1a) and its derivatives Stafib-1 (**2**, Table 1)^15^ and Capstafin^16^ as selective inhibitors of the STAT5b SH2 domain.

**Table 1:** Activities of test compounds against the SH2 domains of STAT5b and STAT5a in fluorescence polarization assays.

No	Structure	STAT5b IC_50_ (µM) or inhibition (%)	STAT5b K_i_ (µM)	STAT5a IC_50_ (µM) or inhibition (%)	STAT5a K_i_ (µM)
**2**		0.154 ± 0.001	0.044 ± 0.001	4.97 ± 0.10	2.42 ± 0.05
**3**		0.19 ± 0.01	0.093 ± 0.003	5.8 ± 0.3	2.9 ± 0.1
**4**		0.082 ± 0.003	0.0088 ± 0.0003	2.7 ± 0.2	1.3 ± 0.1
**5**		0.28 ± 0.01	0.11 ± 0.01	9.7 ± 0.3	4.8 ± 0.1
**6**		0.57 ± 0.02	0.25 ± 0.01	11.6 ± 0.9	5.8 ± 0.4
**7**		no inhibition at 80 µM	—	n. d.	n. d.

n. d.: not determined. Data for **2** are taken from the literature^15^. Mean values ± standard deviations (s.d.) from three independent experiments are given. IC_50_ data were converted to K_i_-values using the published equation^31^.

Despite overwhelming evidence for the biological significance of STAT proteins as therapeutic targets, the development of small-molecule STAT inhibitors has been hampered by the absence of structural characterization of complexes between STATs and small molecules by X-ray crystallography or NMR spectroscopy. The isolated STAT SH2 domains are mostly insoluble; virtually all research studies to date have used STAT constructs containing multiple STAT domains^17, 18^. The molecular weight of the proteins expressed by these constructs is too high for deducing detailed structural information of the STAT-ligand complex by solution-state NMR methods.

We have employed solid-state NMR spectroscopy to verify and characterize the binding of catechol bisphosphate to STAT5b, an interaction which provides the foundation of our docking-based model for the binding of Stafib-1 to STAT5b^15^. Following structure-guided optimization of Stafib-1, we performed a comparative analysis of binding of the optimized inhibitor to wild-type STAT5b protein versus binding to mutant STAT5b proteins using isothermal titration calorimetry (ITC). Here we present the design and binding mode validation of the optimized STAT5b inhibitor Stafib-2, and its prodrug Pomstafib-2, for use as a research tool in cell-based assays.

## Results and Discussion

### Characterization of catechol bisphosphate binding to STAT5 by solid-state NMR

Since binding of **1** to STAT5b Arg618 in the SH2 domain had been indicated in a fluorescence polarization (FP)-based assay^15, ﻿19^, we decided to investigate the mode of STAT5b-binding of **1** using solid-state NMR spectroscopy. The use of solid-state NMR for the characterization of binding between a small molecule and an SH2 domain has not previously been reported. ^13^C direct polarization (DP) / magic-angle spinning (MAS) spectra of ^13^C_6_-**1** revealed that the three ^13^C resonances of ^13^C_6_-**1** split into sets of two in the presence of STAT5b (Fig. 1b). Peak assignment for unbound **1** was carried out on the basis of 2D ^13^C–^13^C correlation spectra of ^13^C_6_-**1** (Supplementary Fig. S1). Similarly, the single ^31^P DP/MAS-NMR resonance of ^13^C_6_-**1** in the absence of STAT5b split into two peaks in the presence of protein, indicative of binding (Fig. 1c). Moreover, the signal width of the two ^31^P-NMR resonance signals was unequal (full widths at half maximum of 94.4 Hz and 147.0 Hz, respectively, Fig. 1c inset). This suggests differences in the rigidity of the chemical environment between the two phosphate groups, indicating that one or more additional amino acids beside Arg618 must contribute to binding of **1**. Inspection of a homology model of the STAT5b SH2 domain^15^ for further putative interaction partners for the phosphate groups suggested Lys600 (Fig. 1d). In fact, a STAT5b Lys600Ala mutant bound with significantly weaker affinity than wild-type STAT5b^15^ to a fluorophore-labelled derivative of **1** in an FP-based assay (Fig. 1e), indicating that STAT5b Lys600 interacts with at least one of the two phosphate groups of **1**. We cannot exclude the possibility that STAT5b amino acid residues other than Arg618 and Lys600 are also involved in binding to **1**.

### Structure-guided optimization of Stafib-1

**Figure 2:**
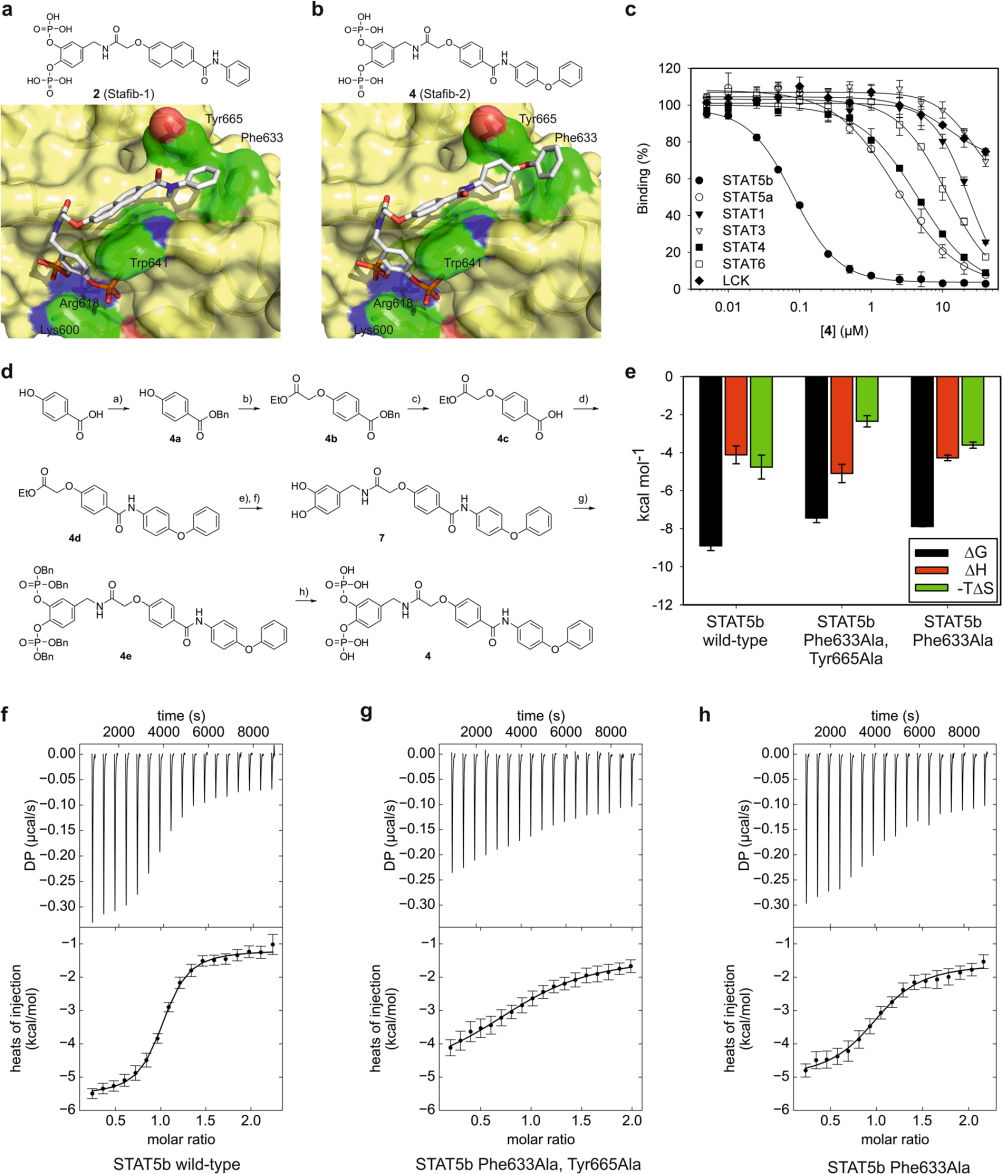
Synthesis and functional characterization of Stafib-2 (**4**). (**a**) Docking pose of Stafib-1 (**2**)^15^. (**b**) Docking pose of **4** predicted by AutoDock Vina. (**c**) Specificity profile of **4**. Error bars represent standard deviations from three independent experiments. (**d**) Synthesis of **4**. (**a**) BnBr, KHCO_3_, DMF, 4 h, 87%; (**b**) ethyl bromoacetate, K_2_CO_3_, DMF, 1 h, 99%; c) Pd/C, H_2_, EtOH, 1 h, 99%; d) 4-phenoxyaniline, EDC, HOBt, DMF, 16 h, 85%; (**e**) 1 M NaOH, THF, 1 h; (**f**) 4-(aminomethyl)benzene-1,2-diol, EDC, HOBt, TEA, DMF, 16 h, 60% over 2 steps; (**g**) dibenzylphosphite, CCl_4_, DIEA, DMAP, 1 h, 78%; (**h**) Pd/C, H_2_, EtOH, 1 h, 94%. (**e**) Thermodynamic parameters extracted from ITC experiments with STAT5b wild-type (n = 3), STAT5b Phe633Ala, Tyr665Ala (n = 3), and STAT5b Phe633Ala (n = 2). Error bars represent standard deviations (s.d.). (**f**) Representative ITC data for binding between **4** and wild-type STAT5b, (**g**) STAT5b Phe633Ala, Tyr665Ala, and h) STAT5b Phe633Ala.

After confirming binding of **1** to the phosphotyrosine binding site of the STAT5b SH2 domain, we aimed to exploit the docking-based binding pose for structure-guided optimization of the catechol bisphosphate derivative Stafib-1 (**2**, Table 1). The terminal phenyl ring of Stafib-1 is predicted to reside in a hydrophobic pocket delineated by the side chains of Phe633 and Tyr665 (Fig. 2a and Supplementary Fig. S2). The distance and the angle between these two aromatic side chains suggested to us that this pocket would be ideally occupied by two aromatic rings connected via an sp^3^-hybridized centre. An obvious chemical moiety is the phenoxyphenyl group, which can be generated by adding a *p*-phenoxy substituent to the terminal phenyl ring of **2**. While the *p*-phenoxy-substituted Stafib-1 derivative **3** was 2-fold less active than **2** (K_i_ = 0.093 ± 0.003 µM for **3** vs. 0.044 ± 0.001 µM for **2**, Table 1), we attributed the reduced activity to inappropriate positioning of the added phenoxy group owing to excessive space requirements of **3** in the protein binding pocket. To facilitate correct placement of the phenoxyphenyl group in its putative binding pocket, we replaced the central naphthyl group of **3** with a phenyl group, resulting in compound **4** (Table 1). Docking of **4** into the STAT5b SH2 domain with AutoDock Vina^20^ suggested a tight fit of the phenoxyphenyl group in the binding pocket delineated by Phe633 and Tyr665, along with face-to-face π-stacking interactions between the side chains of the aromatic amino acids and the phenoxyphenyl group (Fig. 2b). **4** displayed a K_i_ value of 0.0088 ± 0.0003 µM, which represents a more than 10-fold reduction in K_i_ value relative to **3**, and a 5-fold reduced K_i_ value relative to **2**. **4** was found to be highly selective for STAT5b over STAT5a (K_i_ = 1.3 ± 0.1 µM) and other STAT family proteins, as well as the more distantly related SH2 domain of the tyrosine kinase Lck^21^ (Fig. 2c, Table 1). Synthesis of **4** (dubbed Stafib-2) was carried out in 8 synthetic steps in an overall yield of 32% (Fig. 2d). Deletion of the terminal phenoxy group (compound **5**) or the central phenyl amide group (compound **6**) strongly reduced activity (Table 1), consistent with specific protein recognition by **4**. Although **4** has an extremely high affinity for STAT5b, this affinity is crucially dependent on the bisphosphorylated catechol core, as indicated by the lack of activity of the unphosphorylated derivative **7** (Table 1).

### Validation of the docking-based binding mode of Stafib-2 to STAT5b by ITC

In order to verify that the pocket delineated by Phe633 and Tyr665 is indeed involved in binding **4**, we investigated the protein-ligand interaction using isothermal titration calorimetry (ITC). Injection of **4** into a solution of wild-type STAT5b revealed tight binding (K_d_ = 0.32 ± 0.11 µM) with both enthalpic (ΔH = −4.1 ± 0.5 kcal mol^−1^) and entropic contributions (−TΔS = −4.8 ± 0.6 kcal mol^−1^), consistent with binding mediated by both electrostatic and hydrophobic interactions (Fig. 2e,f)^22^. In contrast, binding of **4** to a STAT5b Phe633Ala, Tyr665Ala double mutant was over tenfold weaker (K_d_ = 3.8 ± 1.7 µM, Fig. 2e,g). Of note, while the change in enthalpy was comparable (ΔH = −5.1 ± 0.5 kcal mol^−1^), the entropic contribution of double-mutant binding (−TΔS = −2.4 ± 0.3 kcal mol^−1^) is only half as strong as it is for binding of **4** to wild-type STAT5b. Alanine mutation of STAT5b Phe633 alone was less detrimental for binding (K_d_ = 1.65 ± 0.02 µM, Fig. 2e,h), with a virtually identical change in enthalpy (ΔH = −4.3 ± 0.2 kcal mol^−1^), but a lesser gain in entropy (−TΔS = −3.6 ± 0.2 kcal mol^−1^) as compared to wild-type. These data confirm that both Phe633 and Tyr665 are involved in the interaction. Mutation of Arg618 (K_d_ = 5.6 ± 1.7 µM, Supplementary Fig. S3) or Lys600 (K_d_ = 23 ± 10 µM, Supplementary Fig. S3) to alanine also reduced binding affinity, consisted with the docking pose (Fig. 2b). Binding of **4** to STAT5a demonstrated an approximately 20-fold lower affinity (K_d_ = 6.1 ± 3.2 µM, Supplementary Fig. S3), confirming the specificity of **4** for STAT5b observed in the competitive binding assays. This represents the first time that the binding site of a small-molecule ligand of a STAT protein has been characterized with amino acid resolution by functional comparison of wild-type versus mutant proteins using ITC. Of note, studies carried out on the close homologue STAT5a have shown the hydrophobic pocket delineated by Phe633 and Tyr665 to be important for STAT5a activity^23^, consistent with the likelihood of a functional relevance for the corresponding hydrophobic Stafib-2 binding pocket of STAT5b.

### Validation of Pomstafib-2 in cell-based assays

**Figure 3:**
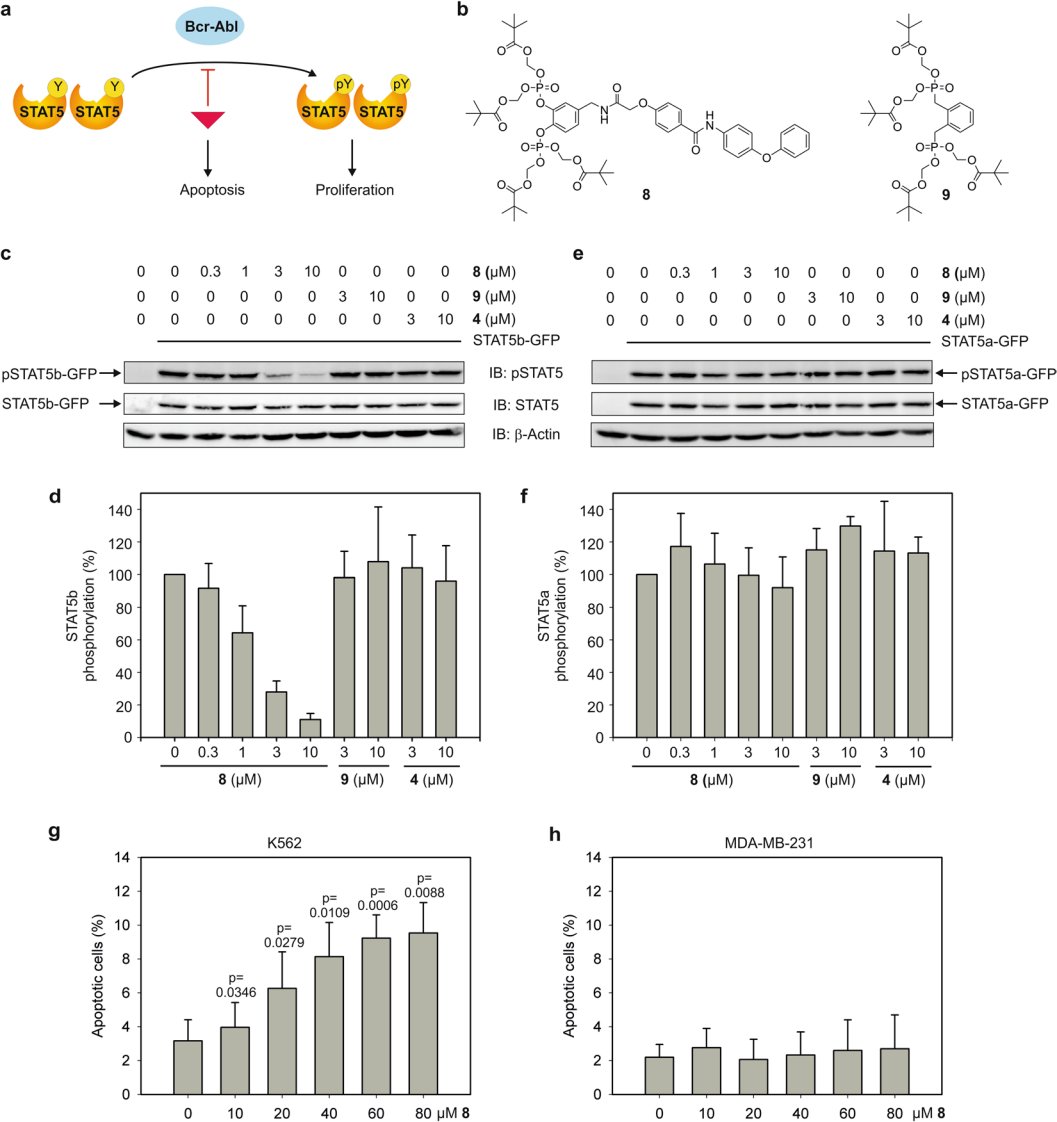
Pomstafib-2 (**8**) inhibits STAT5b signalling with high activity and selectivity in K562 leukaemia cells. (**a**) Expression of Bcr-Abl results in constitutive activation of STAT5. Inhibition of signalling via STAT5 leads to apoptosis. (**b**) Structures of pivaloyloxymethylesters **8** and **9** ^15^. (**c**) Dose-dependent inhibition of STAT5b phosphorylation in STAT5b-GFP-transfected K562 cells by **8**. Cropped blots are displayed; full-length blots are presented in Supplementary Fig. S9. (**d**) Quantitation of the data shown in c) from 2–4 independent experiments, with phospho-STAT5b-GFP levels normalized against total STAT5b-GFP. Error bars represent standard deviations (s.d.). (**e**) Phosphorylation of STAT5a in STAT5a-GFP-transfected K562 cells is not inhibited by **8**. Cropped blots are displayed; full-length blots are presented in Supplementary Fig. S9. (**f**) Quantitation of the data shown in (**e**) from 2–4 independent experiments, with phospho-STAT5a-GFP levels normalized against total STAT5a-GFP. Error bars represent standard deviations (s.d.). (**g**) **8** induces a dose-dependent increase in the apoptotic rate of STAT5-dependent K562 cells. Error bars represent standard deviations (n = 3). Numbers on top of the bars indicate the p-values (t-test, two-tailed, paired). (**h**) **8** does not increase the apoptotic rate of STAT5-independent MDA-MB-231 cells (n = 3).

Human K562 leukaemia cells harbour the tyrosine kinase Bcr-Abl, which leads to constitutive activation of STAT5 (Fig. 3a). In order to test whether the selective activity of Stafib-2 (**4**) also extends to STAT5b in living cells, we masked its negative charges by conversion to the pivaloyloxymethyl ester **8**, which was dubbed Pomstafib-2 (Fig. 3b). Pivaloyloxymethylesters have been successfully shown to mediate cell-permeability of organic phosphates and phosphonates^24–28^. In cells, the pivaloyloxymethyl groups of **8** are cleaved off by intracellular esterases, thereby releasing the parent compound, along with formaldehyde and pivalic acid. Because commercially available antibodies do not allow for distinction between STAT5a phosphorylated at Tyr694 and STAT5b phosphorylated at Tyr699, we used an assay system based on fusion proteins of the individual STAT5 proteins with GFP, which are also recognized by anti-phospho-STAT5 antibodies, but can be distinguished from endogenous STAT5 by their higher molecular weight^15, 16^. Treatment of STAT5b-GFP-transfected K562 cells, in which STAT5 proteins are constitutively activated by Bcr-Abl, with **8** resulted in a dose-dependent decrease in phosphorylation at STAT5b Tyr699, with an IC_50_ of 1.5 µM (Fig. 3c,d). In contrast, phosphorylation of STAT5a at Tyr694 was not significantly reduced by **8** in STAT5a-GFP transfected cells (Fig. 3e,f). Activity of **8** against STAT5b phosphorylation is observed as early as 1 h after addition to the cells, with maximum activity between 2 h and 4 h, and is still significant after 8 h of exposure (Supplementary Fig. S4). **8** is 2.5-fold more active against STAT5b phosphorylation than the pivaloyloxymethylesters of **2** (IC_50_ = 3.8 µM)^15^ and of Capstafin^16^ (IC_50_ = 4.1 µM) (Supplementary Fig. S5). The lack of inhibition of STAT5a/b phosphorylation by the pivaloyloxymethyl ester **9** (Fig. 3c–f), which is based on a phosphonate previously shown to be inactive against STAT5a/b^15^, indicates that neither the formaldehyde nor the pivalic acid released during prodrug cleavage contribute to the inhibitory effect. The unmasked bisphosphate **4** did not show significant activity in cells (Fig. 3c–f), indicating poor cell permeability. Phosphorylation of endogenous STAT5 in the STAT5a/b-GFP transfected cells was also inhibited by **8**, but to a lesser extent than transfected STAT5b-GFP, which presumably reflects the relative abundances of STAT5b and STAT5a in the cells (Supplementary Fig. S6). Consistent with previous observations^15^, these data imply that the majority of phosphorylated endogenous STAT5 protein in K562 cells is STAT5b.

Inhibition of signalling via STAT5b in K562 cells has previously been shown to reduce cell viability and induce apoptosis (Fig. 3a)^4, 5, 29^. Treatment of untransfected K562 cells with **8** increased the apoptotic rate of K562 cells in a dose-dependent manner (Fig. 3g, Supplementary Fig. S7). To verify that the effects of **8** were mediated by inhibition of STAT5 proteins, we carried out the same experiment with MDA-MB-231 cells, which do not harbour constitutively activated STAT5^16^ and therefore can be assumed to grow STAT5-independently. The apoptotic rate of MDA-MB-231 cells was not increased by **8** (Fig. 3h, Supplementary Fig. S8), consistent with the effects on K562 cells being mediated by inhibition of STAT5 proteins. The observation that higher concentrations of **8** are required for induction of apoptosis (Fig. 3g) than for the inhibition of STAT5b phosphorylation (Fig. 3c,d) in K562 cells may reflect the high selectivity of **8** for STAT5b over STAT5a. We hypothesize that uninhibited endogenous, phosphorylated STAT5a could prevent the induction of apoptosis at concentrations of **8** that are already sufficient for selective inhibition of STAT5b phosphorylation.

## Conclusions

We report the rational optimization of the STAT5b SH2 domain inhibitor Stafib-1 (**2**) to Stafib-2 (**4**), which displays significantly increased activity whilst maintaining high selectivity over the closely related SH2 domain of STAT5a. We provide the first application of solid-state NMR for the analysis of ligand binding to an SH2 domain. The high affinity of Stafib-2 for STAT5b allowed us to perform the first comparative analysis of binding between a small-molecule STAT SH2 domain ligand and wild-type versus mutant STAT proteins by ITC, providing experimental validation of the hydrophilic and hydrophobic Stafib-2 binding pockets of STAT5b. The pivaloyloxymethylester Pomstafib-2 (**8**) inhibits STAT5b phosphorylation in cultured human leukaemia cells with an IC_50_ of only 1.5 µM, without significantly affecting the phosphorylation of STAT5a, and increases the apoptotic rate of human leukaemia cells in a STAT5-dependent manner. Since Pomstafib-2 (**8**) currently represents the most active, selective inhibitor of STAT5b activation, we propose its use as a chemical tool to dissect the overlapping and non-redundant functions of the two STAT5 proteins in living cells.

## Methods

### Synthesis of Stafib-2 and Pomstafib-2

#### Tetrabenzyl (4-((2-(4-((4-phenoxyphenyl)carbamoyl)phenoxy)acetamido)methyl)-1,2-phenylene) bis(phosphate) (**4e**)

**7** (130 mg, 0.27 mmol) was benzyl phosphorylated according to Method 3 (Supplementary Information). Purification of the crude material by column chromatography (10% → 20% acetone in DCM) afforded **4e** as colorless oil (210 mg, 78%); R_f_ = 0.26 (CH_2_Cl_2_/acetone 9:1); ^1^H NMR (400 MHz, CDCl_3_) δ = 4.37 (d, *J* = 6.1, 2 H), 4.46 (s, 2 H), 5.05 (dd, *J* = 8.0, 1.9, 8 H), 6.78 (d, *J* = 8.8, 2 H), 6.88–7.04 (m, 6 H), 7.04–7.13 (m, 1 H), 7.12–7.50 (m, 24 H), 7.61–7.76 (m, 2 H), 7.82 (d, *J* = 8.8, 2 H), 8.91 (s, 1 H); ^13^C NMR (101 MHz, CDCl_3_) δ = 42.19, 67.54, 70.31 (d, *J* = 5.8), 70.44 (d, *J* = 6.1), 114.42, 118.49, 119.56, 120.99, 121.02, 121.91, 122.23, 123.05, 125.45, 128.05, 128.08, 128.70, 128.81, 129.03, 129.68, 129.79, 134.37, 135.15, 135.22, 136.18, 140.78 (t, *J* = 6.3), 141.43 (t, *J* = 6.6), 153.31, 157.76, 159.63, 165.44, 168.17; ^31^P NMR (162 MHz, CDCl_3_) δ = −5.9 (s, 1 P), −5.2 (s, 1 P); HRMS (ESI) C_56_H_51_N_2_O_12_P_2_ calculated for [M + H^+^]: 1005.2912; found: 1005.2917.

#### 4-((2-(4-((4-phenoxyphenyl)carbamoyl)phenoxy)acetamido)methyl)-1,2-phenylene bis(dihydrogen phosphate) (4)

Hydrogenation of **4e** (100 mg, 0.1 mmol) according to Method 4 (Supplementary Information) afforded **4** as an off-white solid (60 mg, 94%). Melting point = 136 °C; ^1^H NMR (400 MHz, DMSO-*d*_6_) δ = 4.30 (d, *J* = 5.7, 2 H), 4.66 (s, 2 H), 7.06 (m, 12 H), 7.30 (d, *J* = 8.6, 2 H), 7.39 (t, *J* = 7.9, 2 H), 7.80 (d, *J* = 8.9, 2 H), 7.98 (d, *J* = 8.7, 2 H), 8.75 (t, *J* = 5.9, 1 H), 10.16 (s, 1 H); ^13^C NMR (101 MHz, DMSO) δ = 41.75, 67.43, 114.85, 118.38, 119.72, 121.57, 122.29, 122.47, 123.46, 123.83, 128.07, 129.97, 130.44, 135.68, 136.26, 141.9 (t, *J* = 5.9), 142.8 (t, *J* = 6.0), 152.40, 157.83, 160.75, 165.18, 167.85; ^31^P NMR (162 MHz, DMSO-*d*_6_) δ = −5.9 (s, 1 P), −5.7 (s, 1 P); UV/Vis: λ (nm) = 273, 204; IR (film)${:}\widetilde{\nu }$ = 3419, 3066, 2926, 2853, 2718, 2373, 2348, 2311, 1732, 1716, 1650, 1008, 1541, 1509, 1489, 1456, 1428, 1407, 1349, 1251, 1224, 1181, 1122, 1061, 1022, 1013, 983, 908, 873, 836, 761, 745, 727, 692, 645, 632, 623, 600, 582, 561, 553, 517, 508, 499, 487, 471, 455, 427 cm^−1^; HRMS (ESI) calculated for C_28_H_25_N_2_O_12_P_2_ [M-H^+^]: 643.0888; found: 643.0894.

#### ((((4-((2-(4-((4-Phenoxyphenyl)carbamoyl)phenoxy)acetamido)methyl)-1,2-phenylene)bis(oxy))bis(oxo-l5-phosphanetriyl))tetrakis(oxy))tetrakis(methylene) tetrakis(2,2-dimethylpropanoate) 8

**4** (100 mg, 0.16 mmol) was suspended in dry acetonitrile (5 mL). Diisopropylethyl amine (0.21 ml, 1.28 mmol) and iodomethyl pivalate (300 mg, 1.28 mmol) were added. After stirring at room temperature overnight, the suspension had turned into a clear solution. Volatile components were removed in vacuo. The product was purified by column chromatography (hexane / ethyl acetate 3:1 → 2:1) to yield **8** as a colorless oil (105 mg, 61%); R_f_ = 0.18 (hexane/acetone 2:1); ^1^H NMR (400 MHz, CDCl_3_) δ = 1.2 (d, *J* = 4.8, 36 H), 4.5 (d, *J* = 6.0, 2 H), 4.6 (s, 2 H), 5.7 (t, *J* = 13.3, 8 H), 6.9 (d, *J* = 8.7, 2 H), 7.0 (m, 4 H), 7.1 (m, 3 H), 7.3 (m, 4 H), 7.7 (d, *J* = 8.9, 2 H), 7.9 (d, *J* = 8.7, 2 H), 8.5 (s, 1 H); ^31^P NMR (162 MHz, CDCl_3_) δ = −10.7 (s, 1 P), −9.6 (s, 1 P); ^13^C NMR (75 MHz, CDCl_3_) δ = 26.9, 26.9, 29.8, 38.8, 42.2, 67.7, 83.3 (t, *J* = 4.9), 114.7, 118.5, 119.7, 121.3 (d, *J* = 2.6), 122.1, 122.2, 123.1, 125.9, 129.0, 129.5, 129.8, 134.1, 136.7, 140.2 (t, *J* = 6.5), 140.9 (t, *J* = 6.7), 153.5, 157.7, 159.9, 165.2, 168.1, 176.6, 176.7; UV/Vis: λ (nm) = 273, 205; IR (film)${:}\widetilde{\nu }$ = 3670, 3648, 3626, 3613, 3544, 3481, 3317, 3068, 2976, 2935, 2874, 1767, 1748, 1681, 1668, 1660, 1652, 1606, 1539, 1515, 1505, 1487, 1481, 1463, 1445, 1427, 1406, 1398, 1368, 1311, 1280, 1258, 1169, 1052, 1031, 973, 963, 930, 896, 872, 850, 788, 766, 750, 693, 647, 613, 565, 518, 505, 496, 413 cm^−1^; HRMS (ESI) calculated for C_52_H_67_N_2_O_20_P_2_ [M + H^+^]: 1101.3757; found: 1101.3761.

### MAS NMR Measurements

All NMR experiments were performed at 750.13 MHz for ^1^H, 188.62 MHz for ^13^C and 303.6 MHz for ^31^P on a Bruker AV-750 WB spectrometer (Karlsruhe, Germany) using 4-mm triple-resonance MAS probe (Bruker). Three samples were used in this study: 1) 15 mg of STAT5b with the addition of 0.15 mg of ^13^C_6_-**1**, 2) 10 mg of STAT5b with 0.14 mg of **1** with ^13^C in natural abundance, and 3) 8.25 mg of ^13^C_6_-**1** in the absence of STAT5b. STAT5b was concentrated to a volume of approximately 100 µL by centrifugation using an Amicon^®^ Ultra-15 Centrifugal Filter (molecular weight cut-off 50 kDa, Merck Millipore) at 5000 x g at 4 °C for 6 times, 10 min each. After each centrifugation cycle, the solution was mixed by pipetting to avoid protein precipitation and filter membrane blocking/adsorption. All samples contained the following buffer composition: 100 mM NaCl, 50 mM HEPES pH 7.5, 10% glycerol, 1 mM EDTA, 0.1% NP-40, 1 mM DTT. Each sample was loaded into a 4-mm zirconia MAS rotor with Kel-F spacer, cooled to 228 K (readout temperature) in the magnet and maintained throughout the experiments (±0.3 K). Typical ^13^C and ^31^P *π*-pulse lengths were 8.5 and 7.7 µs, respectively, and ^1^H *π*/2-pulse length was 3.1 µs. ^13^C chemical shifts were externally referenced to the backbone C(O)O^−^ signal of solid glycine·HCl at 176.04 ppm on the TMS scale and ^31^P chemical shifts were referenced to the signal of solid (NH_4_)_2_HPO_4_ at −1.33 ppm on the H_3_PO_4_ scale (85% solution). The MAS rate of all experiments was maintained at 13 kHz ± 5 Hz. The NMR data were processed with Bruker Topspin 3.1 and deconvolution of the ^31^P DP/MAS spectrum of ^13^C_6_-CBP in STAT5b was accomplished with the *dmfit* program^30^.

### Fluorescence polarization (FP) assays

The ability of the test compounds to displace fluorophore-labelled peptides (final concentration: 10 nM) from their respective binding proteins was analysed as previously described^15, 16^. Peptide sequences were: STAT1: 5-carboxyfluorescein-GY(PO_3_H_2_)DKPHVL; STAT3: 5-carboxyfluorescein-GY(PO_3_H_2_)LPQTV-NH_2_; STAT4: 5-carboxyfluorescein-GY(PO_3_H_2_)LPQNID-OH; STAT5a and STAT5b: 5-carboxyfluorescein-GY(PO_3_H_2_)LVLDKW; STAT6: 5-carboxyfluorescein-GY(PO_3_H_2_)VPWQDLI-OH; Lck SH2: 5-carboxyfluorescein-GY(PO_3_H_2_)EEIP. STAT2 was not analysed due to protein instability. Final protein concentrations: STAT1: 420 nM; STAT3: 270 nM; STAT4: 130 nM; STAT5a: 130 nM; STAT5b: 100 nM; STAT6: 310 nM; Lck SH2: 30 nM. These concentrations correspond to the K_d_-values of the respective protein-peptide interactions. Pipetting was carried out in part using a Biomek FX robot (Beckman-Coulter). Proteins and compounds were incubated for 1 h before addition of the fluorescent-labelled peptides. After an additional hour, fluorescence polarization was measured using an Infinite F500 plate reader (Tecan). Final buffer concentrations: 10 mM Tris (pH 8.0), 50 mM NaCl, 1 mM EDTA, 1 mM DTT, 0.1% Nonidet P-40 substitute, 2% DMSO. Changes in FP were converted to percent inhibition based on peptide-protein binding curve fits (SigmaPlot, SPSS Science Software). K_i_-values were calculated from IC_50_ data using the published equation^31^.

### Isothermal titration calorimetry (ITC)

ITC experiments were run on a MicroCal VP-ITC calorimeter. Typical titrations setting were: 25 °C cell temperature, 150 s initial delay, ca. 20 µM STAT protein in 10 mM Tris, 50 mM NaCl, pH 8.0, 200 µM of compound **4** as a tetra sodium salt, stirring speed 300 rpm, reference power to 20 µcal/s. All solutions were degassed before the experiments. The resulting data were analysed by NITPIC^32, 33^ and SEDPHAT^34^ and fitted with the one-site binding model, whilst defining the concentration of **4** as fixed^22^. A low-noise thermogram integration approach was used^22^. Illustrations were made using GUSSI^35^.

### Western Blots

Transfection of cultured K562 cells and Western blotting was performed as previously described^15, 16^. K562 cells were transfected with plasmid encoding either STAT5a-GFP or STAT5b-GFP, using Fugene HD Transfection Reagent (Promega; 1 x 10^6^ cells per well in 1 ml medium with a 4:1 ratio of Fugene:DNA). 24 h later, the cells were treated with compound or DMSO for 4 h (final DMSO concentration: 0.2%). After harvesting, cells were lysed (lysis buffer composition: 50 mM Tris-HCl pH 7.5, 150 mM NaCl, 10 mM Na_4_P_2_O_7_, 10% glycerol, 1% Triton X-100, 1 mM EDTA, 100 ng/mL aprotinin, 1 mM Na_3_VO_4_, 10 mM NaF, 1 mM PMSF). The cell lysate components were separated by SDS-PAGE (10%) and then transferred to a nitrocellulose membrane. Primary antibodies (phospho-STAT5: Cell Signaling #9314; STAT5: Cell Signaling #9363; β-Actin: Cell Signaling #4967) were detected using α-rabbit-HRP secondary antibody (Dako) and ECL (Western Lightning Plus chemiluminescence reagent, Perkin-Elmer), and visualized using an ImageQuant imager (GE Healthcare). ImageJ software (NIH)^36^ was used for signal quantitation.

### Apoptosis assay

Apoptosis assays were performed as previously described^16^. In brief, K562 cells (2.5 × 10^5^ cells per well) or MDA-MB-231 cells (1 × 10^5^ cells per well) were seeded in 24-well tissue culture plates (Corning #3526), and treated with compound **8** at the indicated concentrations (final DMSO concentration: 0.2%) for 48 h. Cells were harvested after 48 h. MDA-MB-231 cells were washed twice with warm phosphate buffered saline (PBS), followed by incubation with Accutase (BD Bioscience) at 37 °C for 10 min. Neutralization of Accutase and cell resuspension was carried out with the cell culture supernatant from each well. After harvesting, cells were centrifuged at 3000 rpm at 4 °C for 5 min, washed twice with cold PBS, and centrifuged again. Cells were stained using the PE Annexin V Apoptosis Detection Kit I (BD Bioscience). Cells were resuspended in binding buffer and incubated with PE Annexin V and 7-AAD at 4 °C for 30 min. Apoptosis was measured using a LSR II flow cytometer (BD Bioscience).

## Electronic supplementary material

Supplementary Info File #1
