# Supplementary material for: Rational development of Stafib-2: a selective, nanomolar inhibitor of the transcription factor STAT5b
Source: Sci Rep. 2017 Apr 11;7:819. doi: 10.1038/s41598-017-00920-3 (PMC5429769; doi:10.1038/s41598-017-00920-3)
Supplement: Supplementary file 1 — Supplementary Info File #1 [file 41598_2017_920_MOESM1_ESM.pdf]

Supplementary Information

Rational development of Stafib-2: a selective, nanomolar inhibitor of the transcription factor STAT5b

Nagarajan Elumalai[1], Angela Berg[1], Stefan Rubner[1], Linda Blechschmidt[1], Chen Song[2,3], Kalaiselvi Natarajan[1], Jörg Matysik[2], and Thorsten Berg[1]*

Table of Contents

Supplementary Table S1 2
Supplementary Figure S1 2
Supplementary Figure S2 3
Supplementary Figure S3 3
Supplementary Figure S4 3
Supplementary Figure S5 4
Supplementary Figure S6 4
Supplementary Figure S7 5
Supplementary Figure S8 5
Supplementary Figure S9 6
Methods 8
Synthetic methods 8
Synthesis and spectroscopic characterization of compounds 9
NMR spectra 17
Supplementary References 20

Supplementary Table S1

Protein	IC_{50} (µM) or maximum inhibition (%)	K_i (µM)
STAT5b	0.082 ± 0.003	0.0088 ± 0.0003
STAT5a	2.7 ± 0.2	1.3 ± 0.1
STAT1	46 ± 2	22.8 ± 1.0
STAT3	30 ± 3 % at 40 µM	n.a.
STAT4	9.1 ± 0.3	4.45 ± 1.49
STAT6	23.7 ± 2.5	11.7 ± 1.2
Lck SH2	25 ± 2 % at 40 µM	n.a.

Supplementary Table S1. Activity profile of **4** in fluorescence polarization assays. n.a.: not applicable. IC_{50} data were converted to K_i-values using the published equation.[1]

Supplementary Figure S1

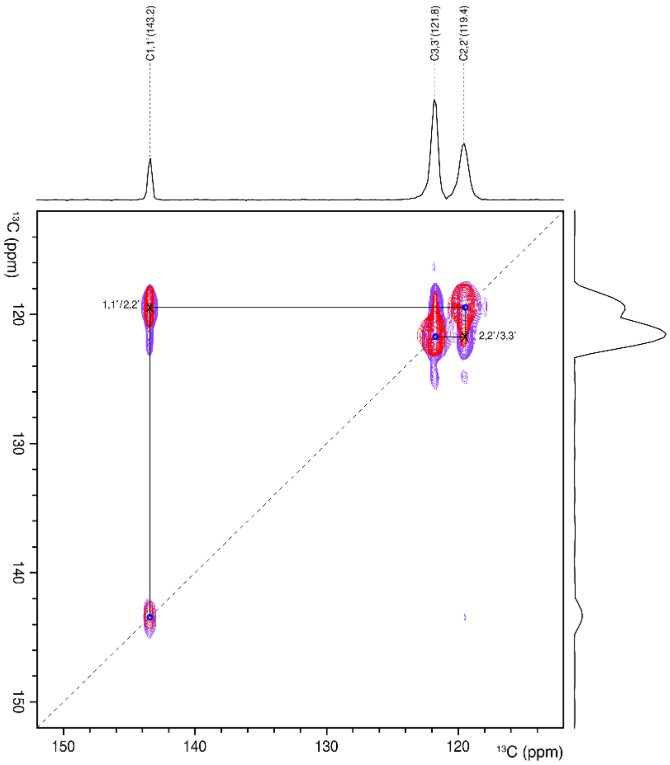

Supplementary Figure S1. Contour plots of the 2D ^{13}C–^{13}C RFDR (radio-frequency-driven dipolar recoupling) MAS NMR spectra of $^{13}C_6$-**1** in HEPES buffer recorded at 228 K using mixing times of 1.82 ms (red) and 3.25 (purple) ms under 13 kHz MAS. Other acquisition and processing parameters are as follows: 96 scans with a recycle delay of 1.5 s for each t_1 point, and a total of 256 points combined in the ω_1-dimension. The vertical and horizontal lines intersecting the diagonal indicate sequences of nearest-neighbor correlations of CBP carbons.

Supplementary Figure S2

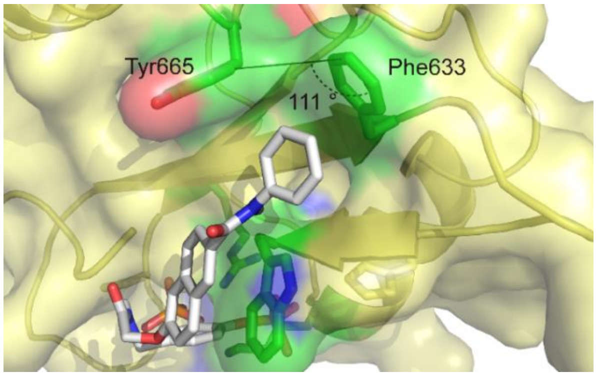

Supplementary Figure S2. Docking pose of Stafib-1[2] in the STAT5b hydrophobic pocket delineated by Tyr665 and Phe633.

Supplementary Figure S3

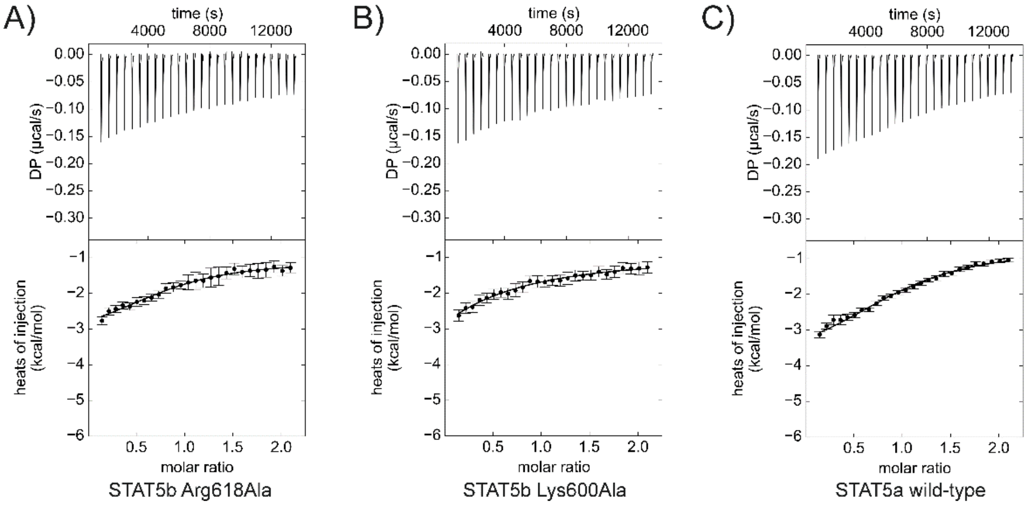

Supplementary Figure S3. Isothermal calorimetry (ITC) analysis of binding between **4** and A) STAT5b Arg618Ala, B) STAT5b Lys600Ala, and C) STAT5a wild-type. Experiments were carried out at least in triplicate; representative examples are shown.

Supplementary Figure S4

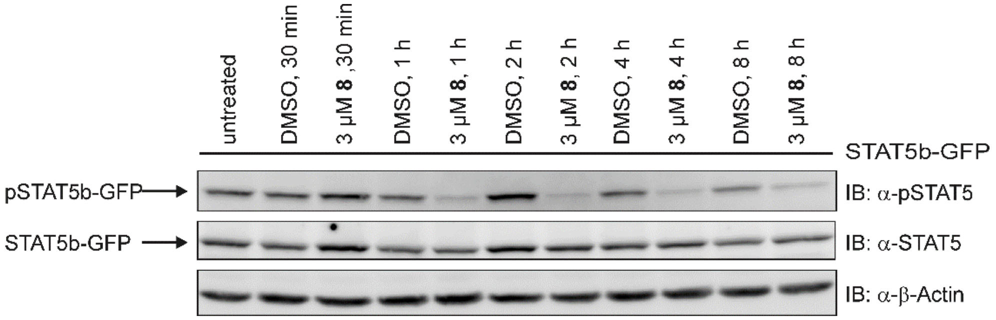

Supplementary Figure S4. Time course of inhibition of STAT5b-GFP phosphorylation by **8**.

Supplementary Figure S5

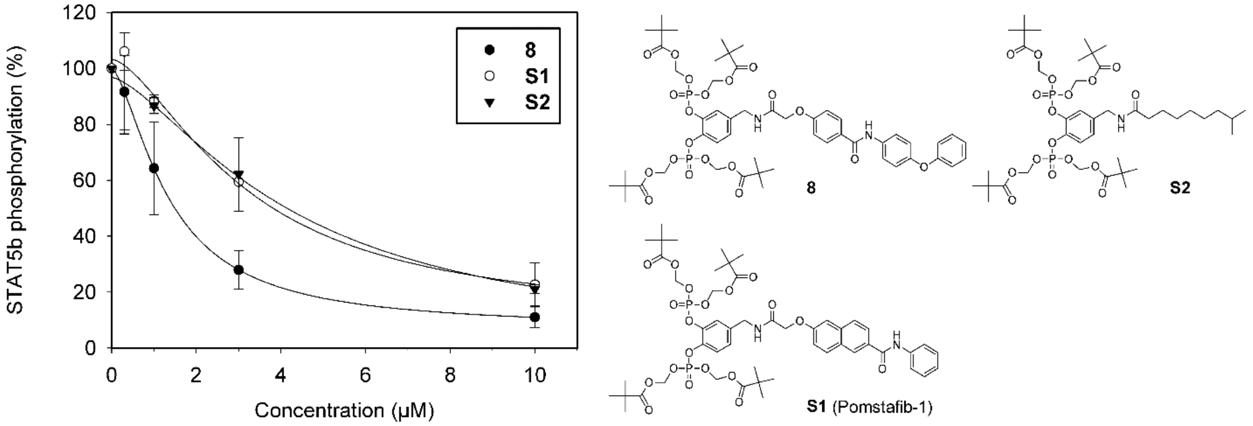

Supplementary Figure S5. Comparison of the activities of pivaloyloxymethylesters **8**, **S1** (Pomstafib-1, the pivaloyloxymethylester of **2**),[2] and **S2** (the pivaloyloxymethylester of Capstafin)[3] against STAT5b phosphorylation in STAT5b-GFP-transfected K562 cells. The data for compounds **S1** and **S2** have been published previously.[2,3]

Supplementary Figure S6

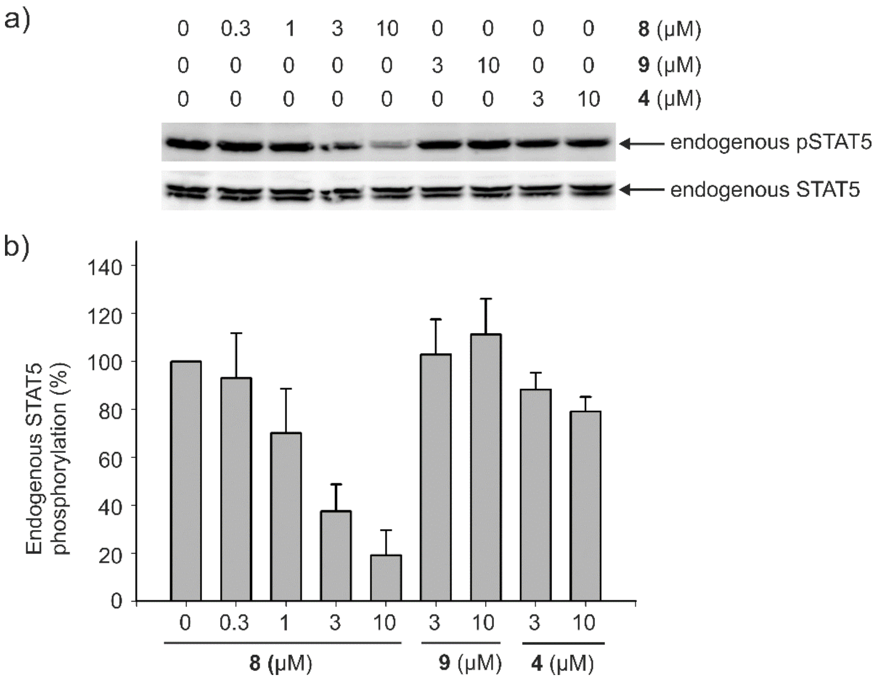

Supplementary Figure S6. a) Effect of the compounds **8**, **9**, and **4** on tyrosine phosphorylation of endogenous total STAT5 in STAT5a/b-GFP-transfected K562 cells. Quantitation of data as shown in a), with phosphorylated STAT5 levels normalized against total STAT5. Error bars represent the standard deviations from at least 4 independent experiments.

Supplementary Figure S7

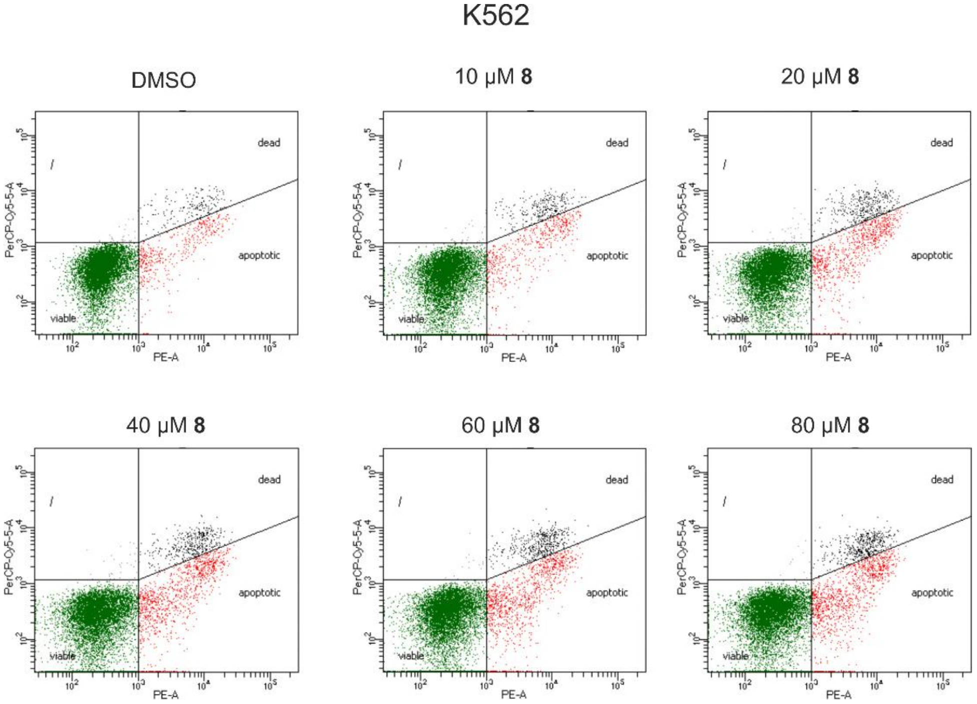

Supplementary Figure S7. Representative results of flow cytometry analysis carried out on K562 cells using the indicated concentrations of **8**. Apoptotic cells, as characterized by Annexin V staining and the absence of 7-AAD staining, are depicted in the lower right-hand section of the flow cytometry plot.

Supplementary Figure S8

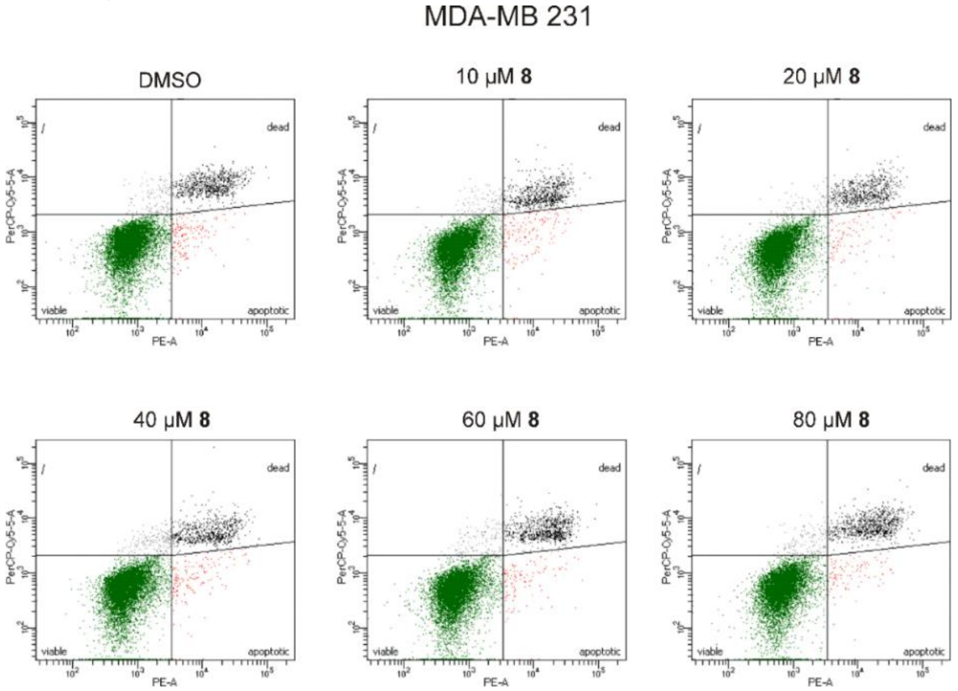

Supplementary Figure S8. Representative results of flow cytometry analysis carried out on MDA MB-231 cells using the indicated concentrations of **8**. Apoptotic cells, as characterized by Annexin V staining and the absence of 7-AAD staining, are depicted in the lower right-hand section of the flow cytometry plot.

Supplementary Figure S9

Full-length blots of cropped blots shown in Fig. 3c and Fig. 3e. The red boxes denote the areas shown in Fig. 3c and 3e. The Bcr-Abl inhibitor Imatinib (right-hand lane) inhibits phosphorylation of both STAT5a and STAT5b.

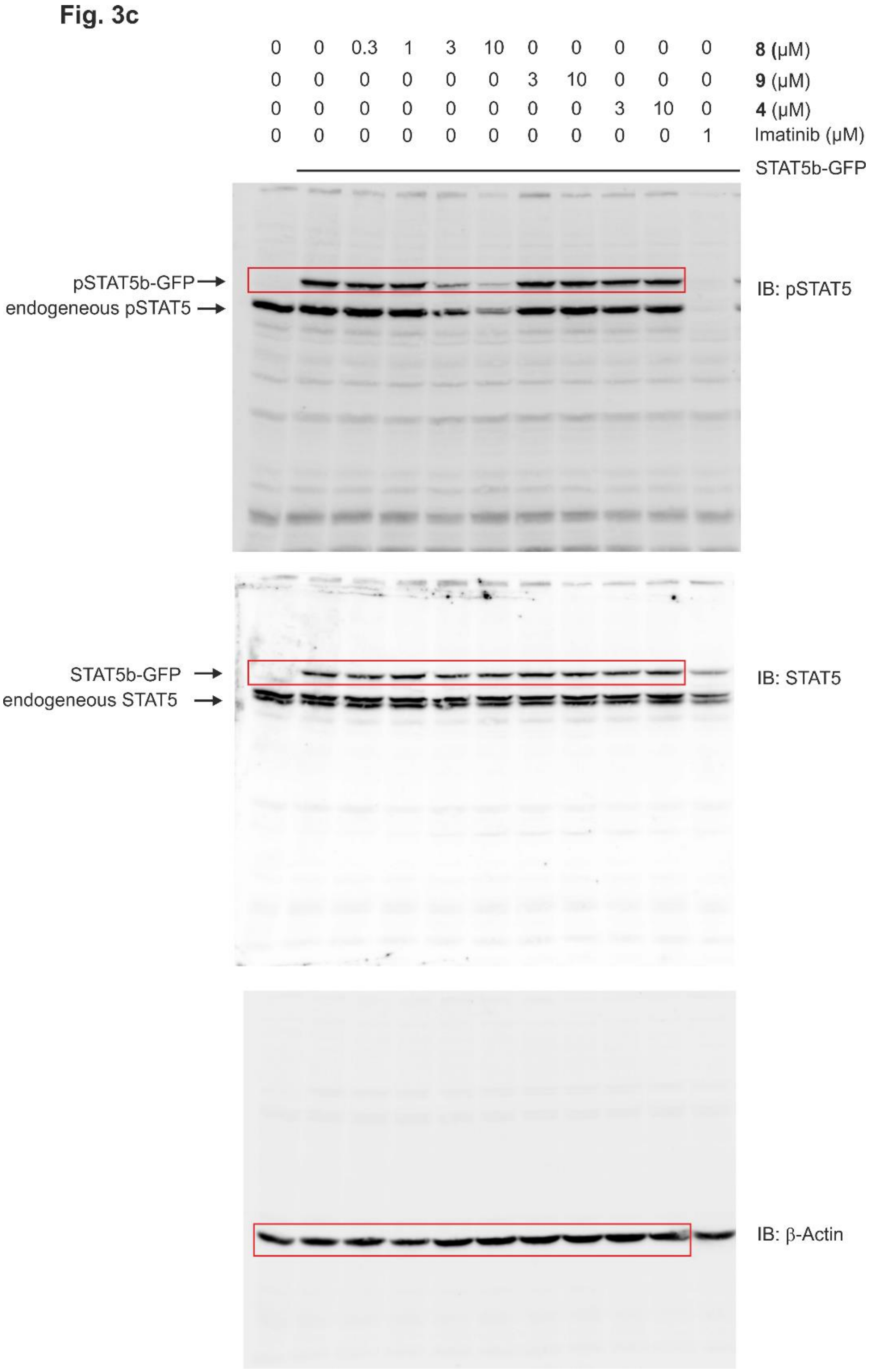

Fig. 3e

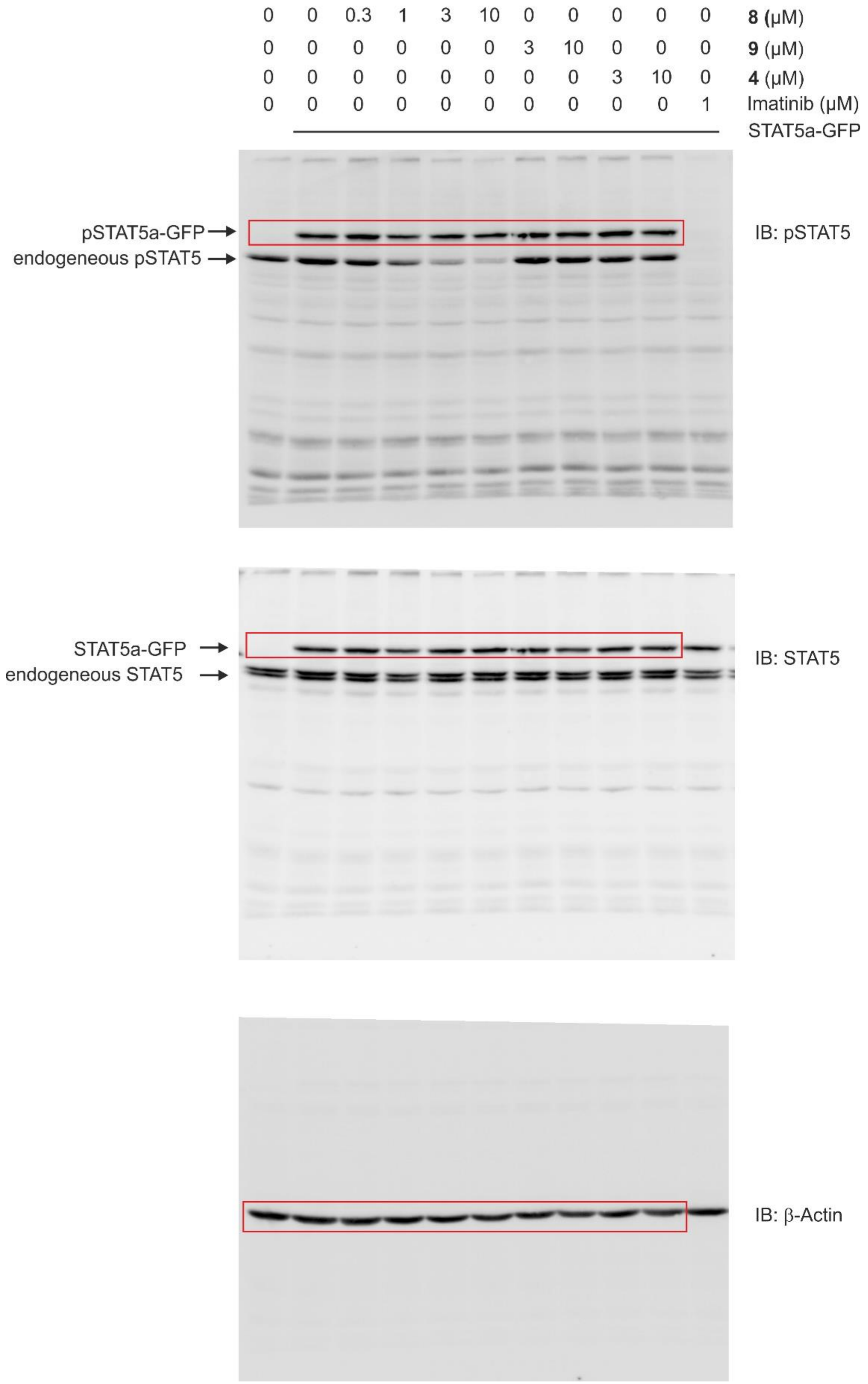
0 0 0.3 1 3 10 0 0 0 0 0 8 (μM)
0 0 0 0 0 0 3 10 0 0 0 9 (μM)
0 0 0 0 0 0 0 0 3 10 0 4 (μM)
0 0 0 0 0 0 0 0 0 0 1 Imatinib (μM)
STAT5a-GFP
pSTAT5a-GFP
endogeneous pSTAT5
IB: pSTAT5
STAT5a-GFP
endogeneous STAT5
IB: STAT5
IB: β-Actin

Methods

Plasmid construction and protein expression

Cloning, expression, and purification protocols for STAT1, STAT3, STAT4, STAT5a, STAT5b, STAT6 and the Lck SH2 domain have been described previously.[2-4] Cloning of the plasmids encoding for STAT5a-GFP and STAT5b-GFP has also been described.[2,3]

Cell culture

Culturing of K562 and MDA-MB-231 cells, which were obtained from the DSMZ (Braunschweig, Germany), was performed as previously described.[2,3] K562 cells were cultured in RPMI 1640 medium, supplemented by 10 % (v/v) FBS, 2 mM L-glutamine, and 1 % (v/v) penicillin/streptomycin, at 37°C, 5% CO_2 and 95% humidity. MDA-MB-231 cells were cultured in Leibovitz's L-15 medium, supplemented with 10 % (v/v) FBS and 1 % (v/v) penicillin/streptomycin, at 37 °C and 95 % humidity with no additional CO_2.

Synthetic methods were applied essentially as previously described.[2,3]

Method 1: Amide coupling

At 0 °C, *N*-(3-dimethylaminopropyl)-*N′*-ethylcarbodiimide hydrochloride (EDC-HCl) (0.1 mmol/mL) was added to a solution of carboxylic acid (0.1 mmol/mL) and 1-hydroxy benzotriazole hydrate (HOBt, 0.1 mmol / mL) in anhydrous DMF. The mixture was stirred for 30 min at 0 °C, followed by 90 min at room temperature. Subsequently, triethylamine (0.3 mmol / mL) and the corresponding amine (0.1 mmol / mL) were added. The mixture was stirred overnight at room temperature. After aqueous work-up and extraction with ethyl acetate, the organic phases were washed with saturated aqueous $NaHCO_3$ solution and dried over Na_2SO_4.

Method 2: Ester hydrolysis

To a solution of ester in THF (ca. 0.2 mmol / mL), approximately the same volume of 1 M NaOH in water was added, followed by stirring at room temperature for 1 h. The reaction was diluted with 50 mL water and acidified to pH 1-2 by addition of 1 N HCl. After extraction of the product with ethyl acetate (3 x 50 mL) and removal of the solvent under reduced pressure, the corresponding carboxylic acid was obtained.

Method 3: Benzyl phosphorylation

To a solution of catechol in dry acetonitrile (0.125 mmol / mL) was added CCl_4 (10 eq), diisopropyl ethylamine (DIPEA) (4 eq) and catalytic amounts of 4-(dimethylamino)pyridine (DMAP). After addition of 3 eq. of dibenzyl phosphite, the mixture was stirred at 0 °C for 30-60 min. Upon completion of the reaction, 0.5 M KH_2PO_4 was added (25 mL / mmol catechol), and the mixture was extracted with ethyl acetate. The combined organic phases were washed with 5% NaCl and H_2O, and were dried over Na_2SO_4. The volatiles were removed under reduced pressure.

Method 4: Hydrogenolysis of benzyl-protected bisphosphates

10% Pd/C (1-2 mg / mol of substrate) was added to a solution of benzyl-protected bisphosphate in absolute ethanol (0.05 mmol / mL) under an argon atmosphere. After exchange of the argon atmosphere for hydrogen, the mixture was stirred for 1-2 h (TLC control). Upon completion of the

reaction, the mixture was filtered through celite, and washed with ethanol. The solvent was removed under reduced pressure. The product was dissolved in water, filtered through cotton, and isolated by lyophilization of the aqueous phase.

Synthesis and spectroscopic characterization of compounds

($^{13}C_6$)-1,2-phenylene tetrabenzyl bis(phosphate) ($^{13}C_6$-**1a**)

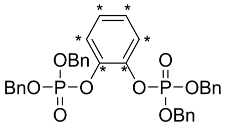

$^{13}C_6$-1a was synthesized from $^{13}C_6$-catechol (30 mg, 0.26 mmol) according to Method 3. The crude product was purified by column chromatography in hexane / ethyl acetate (4:1 → 3:2), and $^{13}C_6$-**1a** was obtained as colorless oil (117 mg, 71%); R_f = 0.32 (hexane/ethylacetate 3:1); ^{1}H NMR (400 MHz, $CDCl_3$) δ=4.93 – 5.21 (m, 8H), 6.74 – 7.00 (m, 1H), 7.06 – 7.41 (m, 22H), 7.46 – 7.66 (m, 1H) ppm; ^{13}C NMR (101 MHz, $CDCl_3$) δ=^{13}C NMR (101 MHz, $CDCl_3$) δ = 70.24 (d, *J*=5.9), 120.14 – 123.08 (m), 124.85 – 127.06 (m), 128.11, 128.68, 128.70, 135.50 (d, *J*=7.4), 140.11 – 142.73 (m) ppm; ^{31}P NMR (162 MHz, $CDCl_3$) δ= -5.27 (s); HRMS (ESI) calculated for $^{12}C_{28}{}^{13}C_6H_{32}O_8NaP_2$ [M+Na$^+$]: 659.1666; found: 659.1664.

1,2-phenylene bis(dihydrogen phosphate) ($^{13}C_6$-**1**)

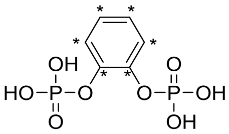

$^{13}C_6$-1 was obtained from **$^{13}C_6$-1a** (100 mg, 0.16 mmol) as an oil according to Method 4. Yield: 42 mg (99%). ^{1}H NMR (400 MHz, CD_3OD) δ=6.93 (s, 1H), 7.17 (s, 1H), 7.33 (s, 1H), 7.57 (s, 1H) ppm; ^{13}C NMR (101 MHz, CD_3OD) δ = ^{13}C NMR (101 MHz, CD_3OD) δ = 122.28 – 124.45 (m), 124.98 – 127.19 (m), 144.28 (dd, *J*=47.0, 27.0) pm; ^{31}P NMR (162 MHz, CD_3OD) δ = -3.41 (s); UV/Vis: λ (nm) = 268, 215; IR (KBr): $\tilde{\nu}$ = 3734, 3720, 3710, 3409, 2337, 1647, 1538, 1662, 1421, 1359, 1283, 1238, 1175, 1110, 1045, 1014, 989, 975, 929, 893, 852, 760, 732, 705, 679, 598, 540, 524, 506, 498, 480, 468, 451, 438, 425 cm^{-1}. HRMS (ESI) calculated for $^{13}C_6H_8NaO_8P_2$ [M+Na$^+$]: 298.9788; found: 298.9786.

6-(2-ethoxy-2-oxoethoxy)-2-naphthoic acid (**3a**)

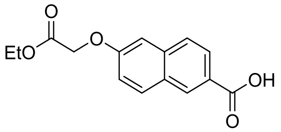

Compound **3a** was synthesized as reported.[2]

Ethyl 2-((6-((4-phenoxyphenyl)carbamoyl)naphthalen-2-yl)oxy)acetate (**3b**)

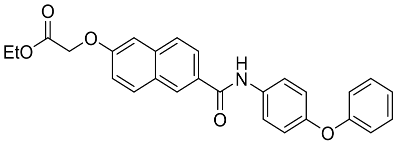

3b was synthesized from **3a** (300 mg, 1.09 mmol) and 4-phenoxyaniline (223 mg, 1.3 mmol) according to Method 1. The crude material was purified by column chromatography (hexane/acetone 3:1) to obtain **3b** as an off-white solid (350 mg, 73%); Melting point: 158-160 °C; R_f = 0.26 (hexane/acetone 3:1); ^{1}H NMR (300 MHz, $CDCl_3$) δ = 1.32 (t, *J*=7.1, 3H), 4.31 (q, *J*=7.1, 2H), 4.74 (s, 2H), 6.91 – 7.18 (m, 6H), 7.21 – 7.28 (m, 1H), 7.33 (td, *J*=7.5, 1.8, 2H), 7.59 – 7.69 (m, 2H), 7.72 (d, *J*=8.6, 1H), 7.79 (d, *J*=9.0, 1H), 7.86 (dd, *J*=8.6, 1.8, 1H), 8.17 (s, 1H), 8.23 – 8.34 (m, 1H); ^{13}C NMR (75 MHz, $CDCl_3$) δ = 14.3, 61.7, 65.5, 107.0, 118.6, 119.7, 119.8, 122.2, 123.2, 124.5, 127.5, 127.6, 128.5, 129.9, 130.4, 130.9, 133.7, 136.1, 153.8, 157.4, 165.9, 165.9, 168.7; HRMS (ESI) calculated for $C_{27}H_{23}NNaO_5$ [M+Na$^+$]: 464.1468; found: 464.1474.

Tetrabenzyl (4-((2-((6-((4-phenoxyphenyl)carbamoyl)naphthalen-2-yl)oxy)acetamido)methyl)-1,2-phenylene) bis(phosphate) (**3c**)

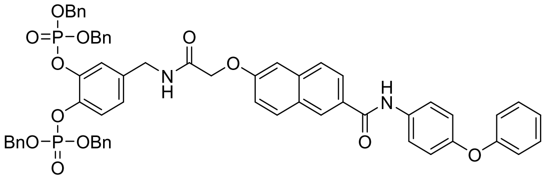

Hydrolysis of the ester functionality of **3b** (340 mg, 0.77 mmol) according to Method 2 provided 302 mg of the corresponding acid. 270 mg (0.65 mmol) of this material was reacted with 4-(aminomethyl)benzene-1,2-diol (144 mg, 0.65 mmol) according to Method 1. Of the 210 mg raw product obtained from this reaction, 190 mg (0.36 mmol) was benzyl phosphorylated according to Method 3. After purification by column chromatography (10% → 15% acetone in DCM), **3c** was obtained as a colorless oil (300 mg, 47 % over three steps); R_f = 0.28 (DCM/acetone 8.5:1.5); ^{1}H NMR (400 MHz, $CDCl_3$) δ = 4.36 (d, *J*=6.1, 2H), 4.59 (s, 2H), 4.94 – 5.11 (m, 8H), 6.73 – 6.85 (m, 1H), 6.94 – 7.01 (m, 4H), 7.01 – 7.16 (m, 6H), 7.16 – 7.35 (m, 22H), 7.61 (d, *J*=8.6, 1H), 7.68 (d, *J*=9.0, 1H), 7.70 – 7.80 (m, 2H), 7.89 (dd, *J*=8.6, 1.8, 1H), 8.29 (d, *J*=2.1, 1H), 9.06 (s, 1H); ^{13}C NMR (101 MHz, $CDCl_3$) δ = 42.01, 67.32, 70.27 (dd, *J*=6.1, 3.4), 107.51, 118.45, 119.01, 119.58, 121.08 (d, *J*=2.3), 121.55 – 121.67 (m), 122.12, 123.05, 124.87, 125.06, 127.30, 127.82, 127.98, 128.01, 128.62, 128.70 – 128.76 (m), 129.77, 131.10, 134.36, 134.87 – 135.42 (m), 135.82, 135.92, 140.59 (t, *J*=6.4), 141.12 (t, *J*=6.7), 153.34, 156.28, 157.69, 166.32, 168.04; ^{31}P NMR (162 MHz, $CDCl_3$) δ = -5.6 (s, 1P), -5.4 (s, 1P); HRMS (ESI) calculated for $C_{60}H_{52}N_2NaO_{12}P_2$ [M+Na$^+$] : 1077.2888; found: 1077.2897.

4-((2-((6-((4-phenoxyphenyl)carbamoyl)naphthalen-2-yl)oxy)acetamido)methyl)-1,2-phenylene bis(dihydrogen phosphate) (**3**)

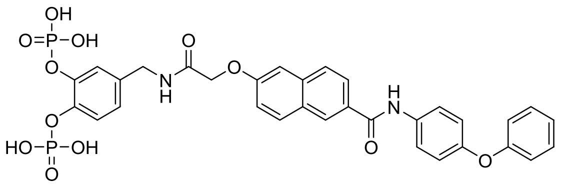

3 was produced from **3c** (250 mg, 0.24 mmol) according to Method 4 as a fluffy solid (150 mg, 91 %); Melting point = 165-67 °C; ^{1}H NMR (400 MHz, DMSO-d_6) δ = 4.30 (d, *J*=6.1, 2H), 4.70 (s, 2H), 6.93 – 7.42 (m, 12H), 7.86 (dd, *J*=30.7, 8.8, 3H), 7.95 – 8.07 (m, 2H), 8.50 (d, *J*=1.9, 1H), 8.76 (t, *J*=6.1, 1H), 10.39 (s, 1H); ^{13}C NMR (101 MHz, DMSO-d_6) δ = 39.64, 41.32, 67.03, 107.28, 117.97, 119.30, 119.62, 122.07, 123.04, 125.07, 126.93, 127.72, 127.84, 130.00, 130.20, 130.64, 135.22, 135.58, 135.69, 141.95, 141.96, 141.99, 142.02, 142.08, 142.86, 142.91, 142.96, 152.07, 157.07, 157.36, 165.47, 167.46; ^{31}P NMR (162 MHz, DMSO-d_6) δ = -4.6 (s, 1P), -4.5 (s, 1P); IR (Film): $\tilde{\nu}$ = 3434, 2925, 1651, 1630, 1539, 1508, 1489, 1431, 1406, 1395, 1272, 1227, 1206, 1173, 1123, 1090, 1073, 1022, 1014, 977, 952, 915, 905, 873, 841, 823, 744, 692, 591, 577, 550, 512, 476, 461 cm^{-1}; HRMS (ESI) calculated for $C_{32}H_{27}N_2O_{12}P_2$ [M-H$^+$]: 693.1045; found 693.1048.

Benzyl 4-hydroxybenzoate (**4a**)

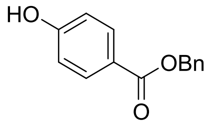

$KHCO_3$ (0.87 g, 8.64 mmol) and benzyl bromide (1.47 g, 8.64 mmol) were added to a solution of 6-hydroxy-2-benzoic acid (1g, 7.2 mmol) in 20 mL dry DMF and stirred at 40 °C for 6 h. Upon completion of the reaction (TLC control), 100 mL of water was added and the mixture was extracted with ethyl acetate (3 x 50 mL). The organic layer was washed with 5% $NaHCO_3$ and dried over Na_2SO_4. Volatiles were removed under reduced pressure. The crude product was purified by column chromatography (hexane/ethyl acetate 9:1 → 3:1) to afford **4a** as an off-white solid (1.44 g, 87%); R_f = 0.34 (hexane/ethylacetate 4:1); Melting point = 110 °C (Lit.: 109.5 °C).[5] ^{1}H NMR (400 MHz, DMSO-d_6) δ = 5.27 (s, 2H), 6.73 – 6.95 (m, 2H), 7.20 – 7.55 (m, 5H), 7.77 – 7.94 (m, 2H), 10.32 (s, 1H); MS (ESI) calculated for $C_{14}H_{12}NaO_3$ [M+Na$^+$]: 251.1; found: 251.1.

Benzyl 4-(2-ethoxy-2-oxoethoxy)benzoate (**4b**)

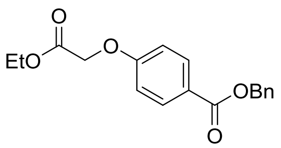

Powdered and dried K_2CO_3 (1.2 g, 8.8 mmol) and ethyl bromoacetate (0.6 mL, 5.26 mmol) were added to the solution of **4a** (1 g, 4.4 mmol) in dry DMF (10 mL) and stirred at room temperature

for 1 h (TLC control). 100 mL of water were added and the mixture was extracted with ethylacetate (3 x 50 mL). The combined organic phases were dried over Na_2SO_4. Volatiles were removed under reduced pressure to afford **4b** as a white solid (1.37 g, 99%); Melting point = 210 °C; ^{1}H NMR (300 MHz, $CDCl_3$) δ = 1.28 (t, *J*=7.1, 3H), 4.25 (q, *J*=7.1, 2H), 4.65 (s, 2H), 5.32 (s, 2H), 6.80 – 7.05 (m, 2H), 7.18 – 7.56 (m, 5H), 8.00 – 8.06 (m, 2H); MS (ESI) calculated for $C_{18}H_{18}NaO_5$ [$M+Na^+$]: 337.1; found: 337.1.

4-(2-ethoxy-2-oxoethoxy) benzoic acid (**4c**)[6]

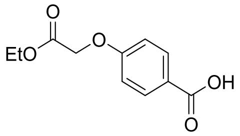

To a solution of **4b** (1.2 g, 3.81 mmol) in ethyl acetate / ethanol (1:1) was added Pd/C (0.12 g) under an argon atmosphere. Argon was replaced by hydrogen. After completion of the reaction in 2 h (TLC control), the mixture was filtered through celite, which was washed with ethanol. Combined filtrates were evaporated to dryness to afford **4c** as an off-white solid (0.85 g, 99.4%); Melting point = 114 °C; ^{1}H NMR (400 MHz, DMSO-d_6) δ = 1.18 (t, *J*=7.1, 3H), 4.15 (q, *J*=7.1, 2H), 4.84 (s, 2H), 6.86 – 7.14 (m, 2H), 7.79 – 8.00 (m, 2H); ^{13}C NMR (101 MHz, DMSO-d_6) δ = 14.01, 60.75, 64.67, 114.35, 123.79, 131.25, 161.09, 166.88, 168.33; HRMS (ESI) calculated for $C_{11}H_{11}O_5$ [$M-H^+$]: 223.0612; found: 223.0612.

Ethyl 2-(4-((4-phenoxyphenyl)carbamoyl)phenoxy)acetate (**4d**)

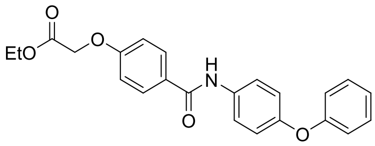

4c (0.2 g, 0.89 mmol) and 4-phenoxyaniline (167 mg, 0.9 mmol) dissolved in 4 mL dry DMF were reacted according to Method 1 (Supplementary Information). The crude material was purified by column chromatography (hexane/ethyl acetate 9:1 → 3:1) to afford **4d** as white solid (290 mg, 85%). R_f = 0.43 (3:1 hexane/ethyl acetate); Melting point = 122 °C; ^{1}H NMR (400 MHz, $CDCl_3$) δ = 1.30 (t, *J*=7.1, 3H), 4.28 (q, *J*=7.1, 2H), 4.67 (s, 2H), 6.90 – 6.96 (m, 2H), 6.96 – 7.03 (m, 4H), 7.05 – 7.12 (m, 1H), 7.28 – 7.36 (m, 2H), 7.54 – 7.61 (m, 2H), 7.78 – 7.85 (m, 2H), 7.94 (s, 1H); ^{13}C NMR (101 MHz, $CDCl_3$) δ = 14.28, 61.72, 65.36, 114.68, 118.57, 119.73, 122.16, 123.20, 128.27, 129.10, 129.84, 133.67, 153.69, 157.64, 160.63, 165.25, 168.49; HRMS (ESI) calculated for $C_{23}H_{21}NNaO_5$ [$M+Na^+$]: 414.1312; found: 414.1311.

4-(2-((3,4-dihydroxybenzyl)amino)-2-oxoethoxy)-N-(4-phenoxyphenyl)benzamide (**7**)

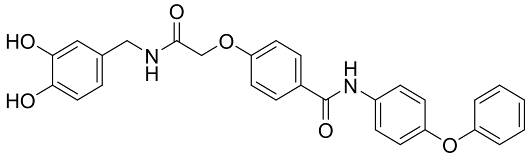

4d (220 mg, 0.56 mmol) was dissolved in 3 mL THF, and 3 mL 1 M NaOH were added to cleave the ester group according to Method 2 (Supplementary Information). Of the 205 mg raw product obtained, 200 mg (0.55 mmol) was reacted with 4-(aminomethyl)benzene-1,2-diol (132 mg, 0.60 mmol) according to Method 1. **7** was obtained as an off-white solid (0.160 g, 60 % over two steps); Melting point = 182 °C; ^{1}H NMR (300 MHz, DMSO-d_6) δ = 4.16 (d, *J*=5.5, 2H), 4.60 (s, 2H), 6.51 (dd, *J*=8.0, 2.0, 1H), 6.58 – 6.75 (m, 2H), 6.90 – 7.18 (m, 7H), 7.36 (tt, *J*=7.5, 2.2, 2H), 7.67 – 7.85 (m, 2H), 7.87 – 8.01 (m, 2H), 8.52 (t, *J*=5.8, 1H), 8.77 (s, 2H), 10.11 (s, 1H); ^{13}C NMR (101 MHz, DMSO-d_6) δ = 41.54, 66.95, 114.34, 115.04, 115.25, 117.88, 118.31, 119.24, 121.96, 122.96, 127.52, 129.44, 129.94, 129.99, 135.18, 144.19, 145.058, 151.91, 157.35, 160.29, 164.68, 166.98; HRMS (ESI) calculated for $C_{28}H_{24}N_2NaO_6$ [M+Na$^+$]: 507.1527; found: 507.1522.

2-(4-(phenylcarbamoyl)phenoxy)acetic acid (**5d**)

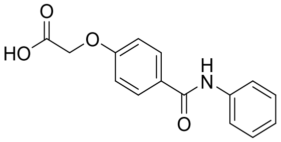

4c (0.2 g, 0.89 mmol) and aniline (0.11 g, 1.16 mmol) in dry DMF were reacted according to Method **1** to yield 0.16 g of raw product. 0.14 g of this material were dissolved in THF (2.5 mL), and an aqueous solution of 1 M NaOH (2.5 mL) was added according to Method 2. The resulting product **5d** (0.145 g, 60% over 2 steps) was obtained as a white solid. Melting point = 216 °C. ^{1}H NMR (400 MHz, DMSO-d_6) δ = 4.77 (s, 2H), 6.88 – 7.14 (m, 3H), 7.18 – 7.50 (m, 2H), 7.74 (dt, *J*=8.8, 1.7, 2H), 7.83 – 8.05 (m, 2H), 10.06 (s, 1H), 13.05 (s, 1H); ^{13}C NMR (75 MHz, DMSO-d_6) δ = 64.51, 114.09, 120.27, 123.40, 127.51, 128.53, 129.48, 139.31, 160.31, 164.85, 169.85. HRMS (ESI) calculated for $C_{14}H_{13}NO_4$ [M-H$^+$]: 270.0772; found: 270.0773.

4-(2-((3,4-Dihydroxybenzyl)amino)-2-oxoethoxy)-N-phenylbenzamide (**5e**)

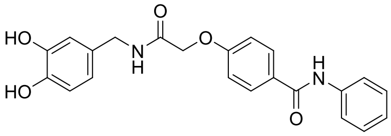

5d (0.1 g, 0.37 mmol) was reacted with 4-(aminomethyl)benzene-1,2-diol (0.082 g, 0.37 mmol) according to Method 1. Purification of the crude product by column chromatography (2% methanol in DCM) afforded **5e** as a white solid (0.13 g, 89 %); R_f = 0.40 (CH_2Cl_2/MeOH 50:1); Melting point

= 220 °C; ^{1}H NMR (400 MHz, DMSO-d_6) δ = 4.18 (d, *J*=6.0, 2H), 4.62 (s, 2H), 6.52 (dd, *J*=8.0, 2.0, 1H), 6.60 – 6.76 (m, 2H), 6.99 – 7.18 (m, 3H), 7.34 (t, *J*=7.9, 2H), 7.65 – 7.83 (m, 2H), 7.86 – 8.07 (m, 2H), 8.54 (t, *J*=6.0, 1H), 8.74 (s, 1H), 8.83 (s, 1H), 10.09 (s, 1H); ^{13}C NMR (101 MHz, DMSO-d_6) δ = 41.55, 66.95, 114.33, 115.05, 115.25, 118.31, 120.28, 123.41, 127.59, 128.53, 129.48, 129.99, 139.29, 144.19, 145.06, 160.29, 164.81, 166.97; HRMS (ESI) calculated for $C_{22}H_{19}N_2O_5$ [M-H$^+$]: 391.1299; found: 391.1301.

Tetrabenzyl (4-((2-(4-(phenylcarbamoyl)phenoxy)acetamido)methyl)-1,2-phenylene) bis(phosphate) (**5f**)

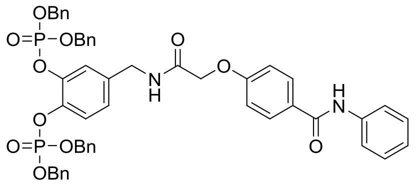

5e (0.1 g, 0.25 mmol) dissolved in dry CH_3CN/DMF (5:1) and benzyl phosphorylated according to Method 3. Purification by column chromatography (20:1 → 9:1 DCM/acetone) provided the product **5f** as a viscous oil (0.198 g, 86%); R_f = 0.28 (CH_2Cl_2/acetone 9:1); ^{1}H NMR (400 MHz, $CDCl_3$) δ = 4.36 (d, *J*=6.1, 2H), 4.42 (s, 2H), 5.04 (dt, *J*=8.4, 1.9, 8H), 6.70 – 6.86 (m, 2H), 6.99 (dd, *J*=8.5, 2.0, 1H), 7.04 – 7.13 (m, 2H), 7.14 – 7.37 (m, 23H), 7.71 (d, *J*=7.2, 1H), 7.76 – 7.90 (m, 2H), 8.86 (s, 1H); ^{13}C NMR (75 MHz, $CDCl_3$) δ = 42.15, 67.44, 70.33 (dd, *J*=8.2, 6.0), 114.41, 120.55, 121.00 (d, *J*=2.5), 121.87 (d, *J*=2.5), 124.15, 125.35, 128.04 (d, *J*=1.9), 128.66, 128.76, 128.89, 129.02, 129.64, 135.14, 135.24, 136.17, 138.73, 140.74 (t, *J*=6.4), 141.40 (t, *J*=6.6), 159.63, 165.47, 168.09; ^{31}P NMR (162 MHz, $CDCl_3$) δ = -5.8 (s, 1P), -5.2 (s, 1P).

4-((2-(4-(phenylcarbamoyl)phenoxy)acetamido)methyl)-1,2-phenylene bis(dihydrogen phosphate) (**5**)

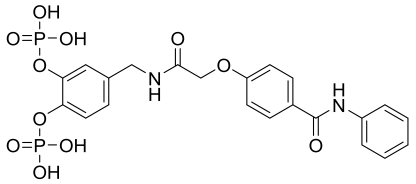

5f (0.15 g, 0.11 mmol) were hydrogenated according to Method 4. Lyophilization yielded a white fluffy solid (0.055 g, 92%); Melting point = 180-182 °C; ^{1}H NMR (400 MHz, DMSO-d_6) δ = 4.61 (s, 2H), 6.75 – 7.47 (m, 7H), 7.66 – 8.10 (m, 4H), 8.66 (s, 1H), 10.15 (s, 1H); ^{13}C NMR (101 MHz, DMSO-d_6) δ = 41.28, 66.90, 114.30, 120.28, 122.05, 122.80, 123.33, 127.59, 128.48, 129.53, 135.03, 139.36, 142.85, 143.80, 160.26, 164.85, 167.28; ^{31}P NMR (162 MHz, DMSO-d_6) δ = -4.5 (s, 1P), -4.1 (s, 1P); UV/Vis: λ (nm) = 268, 204; IR (film): $\tilde{\nu}$ = 3669, 3420, 3323, 2925, 1650, 1607, 1536, 15089, 1441, 1351, 1328, 1285, 1267, 1252, 1225, 1183, 1158, 1121, 1078, 1062, 1023, 977, 893, 849, 823, 761, 729, 692, 629, 581, 515, 505, 455 cm^{-1}; HRMS (ESI) calculated for $C_{22}H_{21}N_2O_{11}P_2$ [M-H$^+$]: 551.0626; found: 551.0620.

2-(4-Phenoxyphenoxy)acetic acid (**6a**)

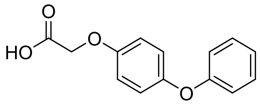

To a solution of 4-phenoxyphenol (0.93 g, 5 mmol) in anhydrous DMF (7 mL) were added K_2CO_3 (1.4 g, 10 mmol) and ethyl bromoacetate (0.56 ml, 5.5 mmol), and the mixture was stirred for 1 h at room temperature (TLC control). 150 mL of distilled water was added, and the mixture was extracted with ethyl acetate (3x50 mL). The combined organic layer was dried over Na_2SO_4, and the volatile components were removed in vacuo. The raw product was dissolved in 20 mL of THF and 20 mL of 1M NaOH was added dropwise at 0 °C. The mixture was stirred for 2 h at room temperature, diluted with 100 mL of water, and extracted with ethyl acetate (50 mL). The aqueous layer was acidified to pH 1.5 and extracted with ethyl acetate (2x50 mL). The combined organic phases were dried over Na_2SO_4, and the volatile components were removed in vacuo to afford **6a** as a white solid (1.21 g, 99 % over two steps), which was used in the next step without additional purification. ^{1}H NMR (300 MHz, DMSO-*d*$_6$) δ = 4.64 (s, 2H), 6.83 – 7.00 (m, 6H), 7.05 (t, *J*=7.4, 1H), 7.23 – 7.45 (m, 2H), 12.97 (bs, 1H); ^{13}C NMR (75 MHz, DMSO-*d*$_6$) δ = 64.89, 115.73, 117.37, 120.50, 122.63, 129.85, 149.80, 154.05, 157.81, 160.59, 170.17; MS (ESI) calculated for $C_{14}H_{11}O_4$ [M-H$^+$]: 243.0663; found: 243.0660.

Tetrabenzyl (4-((2-(4-phenoxyphenoxy)acetamido)methyl)-1,2-phenylene) bis(phosphate) (**6b**)

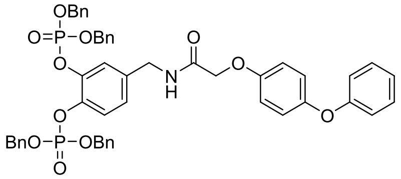

6a (200 mg, 0.78 mmol) was reacted with 4-(aminomethyl)benzene-1,2-diol (180 mg, 0.78 mmol) according to Method 1 to obtain the corresponding amide (205 mg) as a raw product. 150 mg of the amide was benzyl phosphorylated according to Method 3. The crude product was purified by column chromatography (9:1 DCM/acetone) to afford **6b** as a colorless oil (304 mg, 61% over two steps); R_f = 0.30 (CH_2Cl_2/acetone 9:1); ^{1}H NMR (400 MHz, $CDCl_3$) δ = 4.40 (d, *J*=6.0, 2H), 4.47 (s, 2H), 4.93 – 5.17 (m, 8H), 6.79 – 7.09 (m, 9H), 7.24 (dddd, *J*=13.2, 8.8, 5.6, 1.6, 24H); ^{13}C NMR (75 MHz, $CDCl_3$) δ = 42.20, 68.03, 70.29 (dd, *J*=5.8, 3.8), 116.01, 118.11, 120.86, 121.18 (d, *J*=2.5), 122.04 (d, *J*=2.7), 122.97, 125.11, 128.12 (d, *J*=2.1), 128.67, 128.73, 129.82, 135.36, 135.46, 135.86, 140.64 – 141.08 (m), 141.31 – 141.66 (m), 151.72, 153.31, 158.06, 165.94, 168.22; ^{31}P NMR (162 MHz, $CDCl_3$) δ = -5.28 (s, 1P), -5.14 (s, 1P); HRMS (ESI) calculated for $C_{49}H_{46}NO_{11}P_2$ [M+H]$^+$: 886.2541; found: 886.2539.

4-((2-(4-phenoxyphenoxy)acetamido)methyl)-1,2-phenylene bis(phosphate) (**6**)

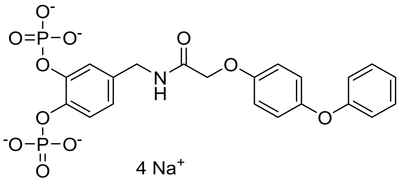

6b (280 mg, 0.53 mmol) was debenzylated according to Method 4 to afford the phosphoric acid ester (120 mg) as a white powder. This was dissolved in 3 mL H_2O and mixed with an aqueous solution of $NaHCO_3$ (78 mg, 0.93 mmol, 4 eq.). The clear solution was filtered through cotton (5 cm in a glass pipette), and the cotton was washed with 4 mL of H_2O and lyophilized again to afford **6** as an off-white solid (131mg, 91%); Decomposition point > 150 °C; ^{1}H NMR (400 MHz, D_2O) δ = 4.27 (s, 2H), 4.53 (s, 2H), 6.74 (d, *J*=8.3, 1H), 6.92 (q, *J*=9.0, 6H), 7.04 (t, *J*=7.4, 1H), 7.26 (q, *J*=9.7, 8.5, 4H); ^{13}C NMR (101 MHz, D_2O) δ = 42.66, 67.39, 116.23, 118.03, 119.83 (d, *J*=2.3), 120.46 (d, *J*=2.5), 120.87, 120.93, 123.49, 130.18, 131.25, 143.86 – 144.04 (m), 144.50 – 144.66 (m), 150.89, 153.72, 157.69, 171.17; ^{31}P NMR (162 MHz, D_2O) δ = 1.55; UV/Vis: λ (nm) = 271, 203; IR (film): $\tilde{\nu}$ = 3750, 3734, 3723, 3710, 3688, 3408, 2547, 2444, 1923, 1770, 1731, 1665, 1590, 1542, 1505, 1489, 1454, 1427, 1403, 1350, 1338, 1287, 1220, 1109, 994, 959, 875, 833, 750, 694, 658, 585, 528, 510, 499, 481, 422 cm^{-1}; HRMS (ESI) calculated for $C_{21}H_{20}NO_{11}P_2$ [M-4 Na^++3H^+] : 524.0517; found: 524.0514.

NMR spectra

^{1}H NMR of compound **4**

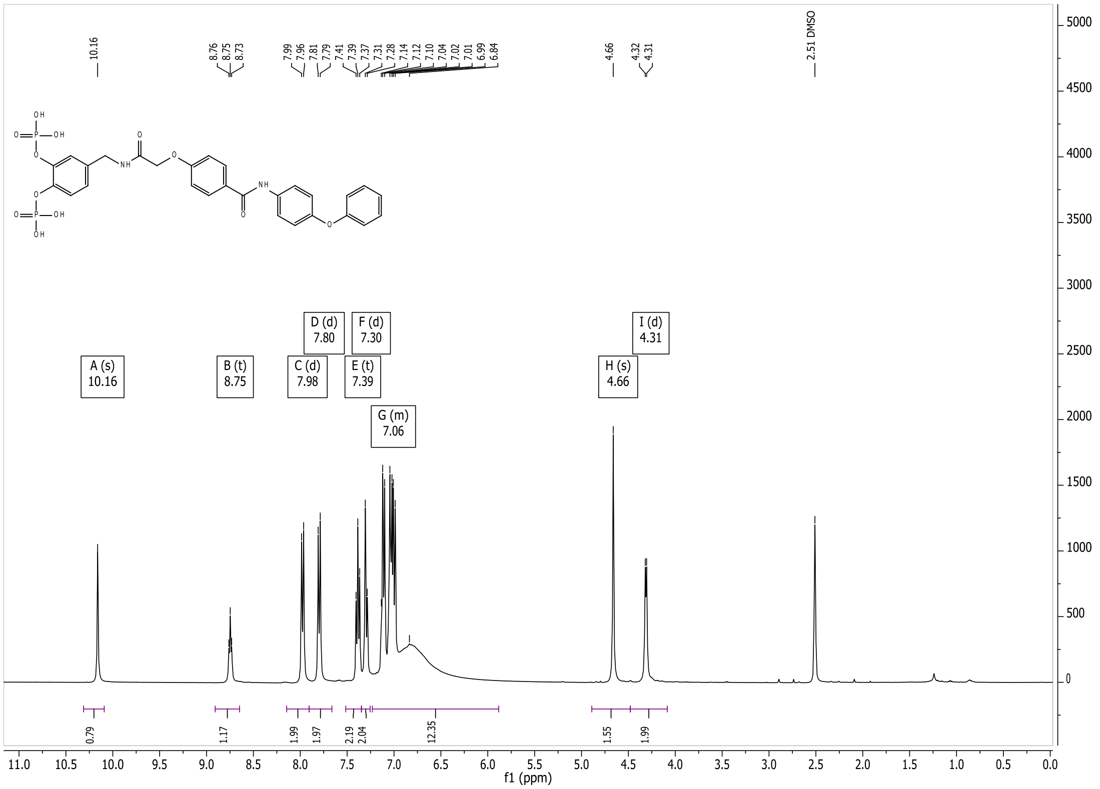

^{13}C NMR of compound **4**

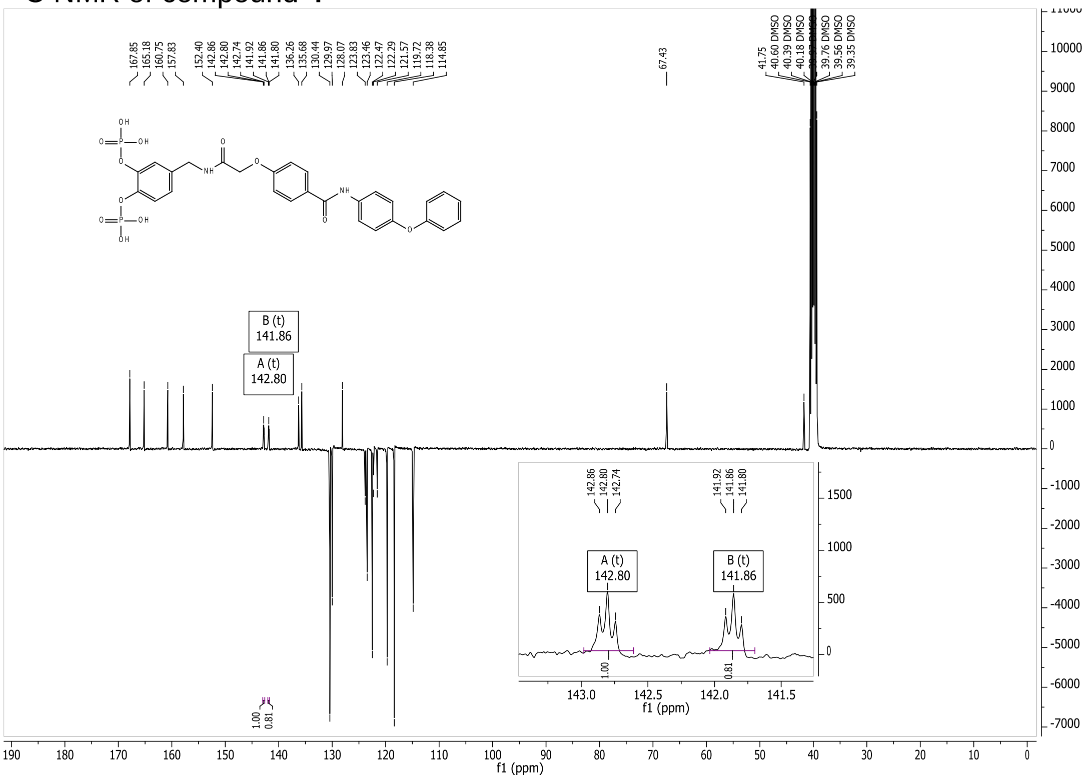

^{31}P NMR of compound **4**

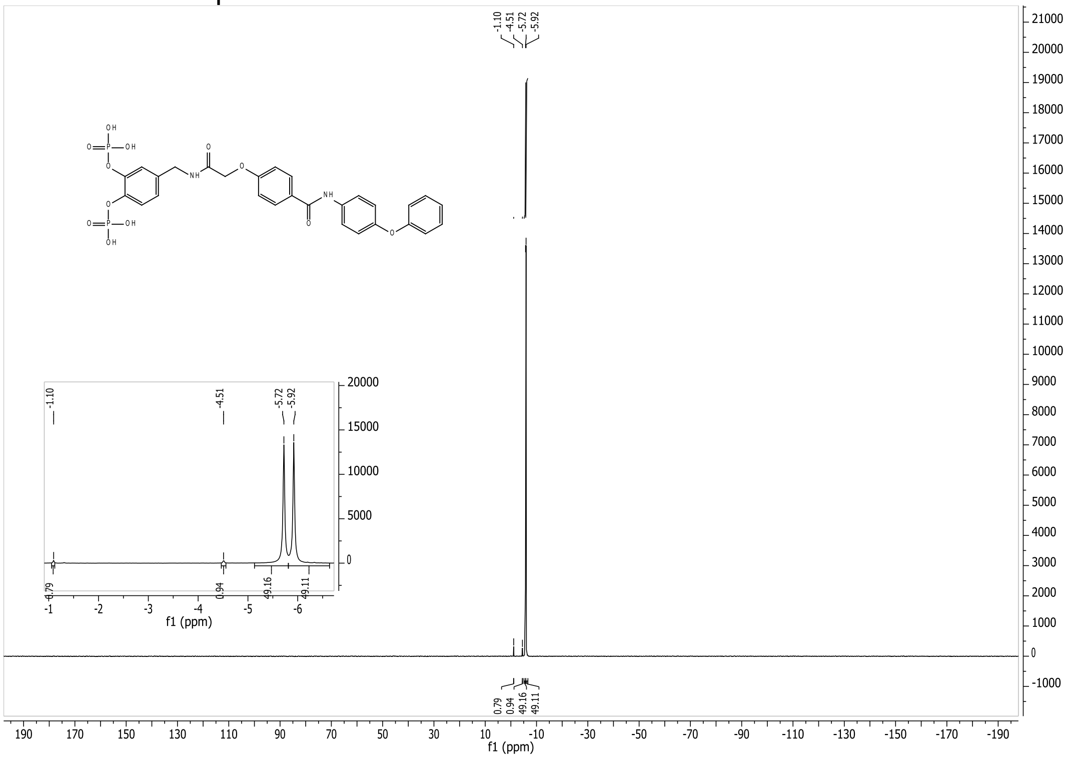

^{1}H NMR of compound **8**

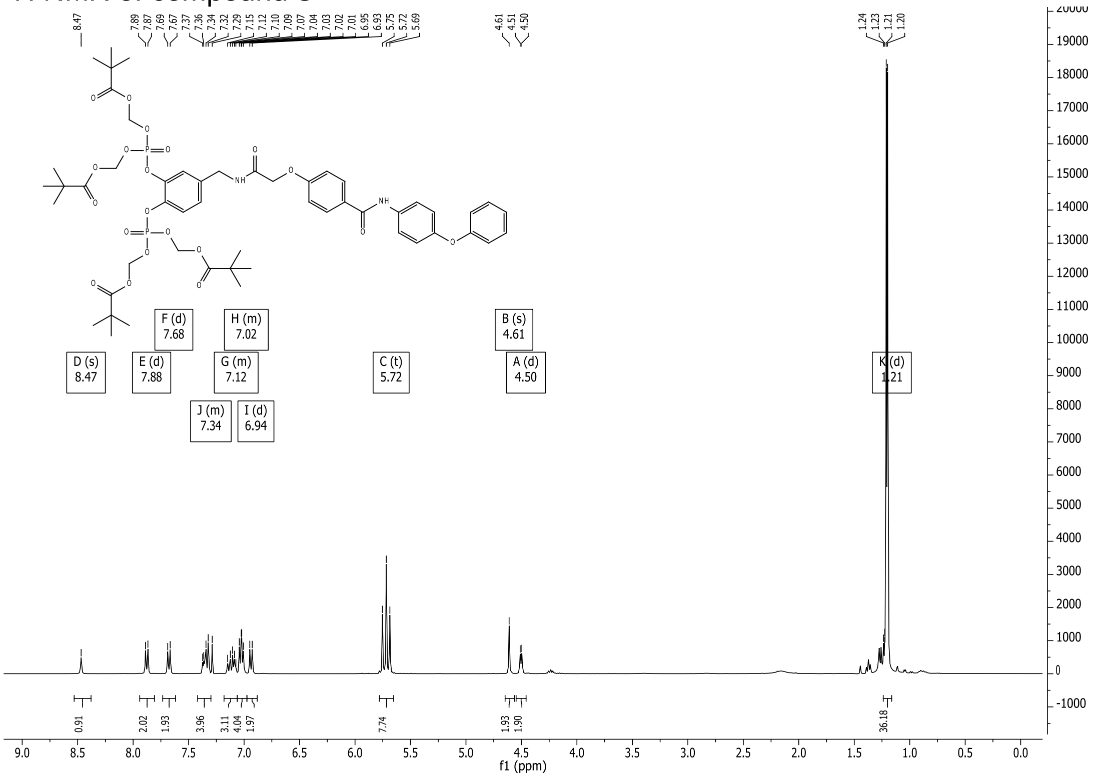

^{13}C NMR of compound **8**

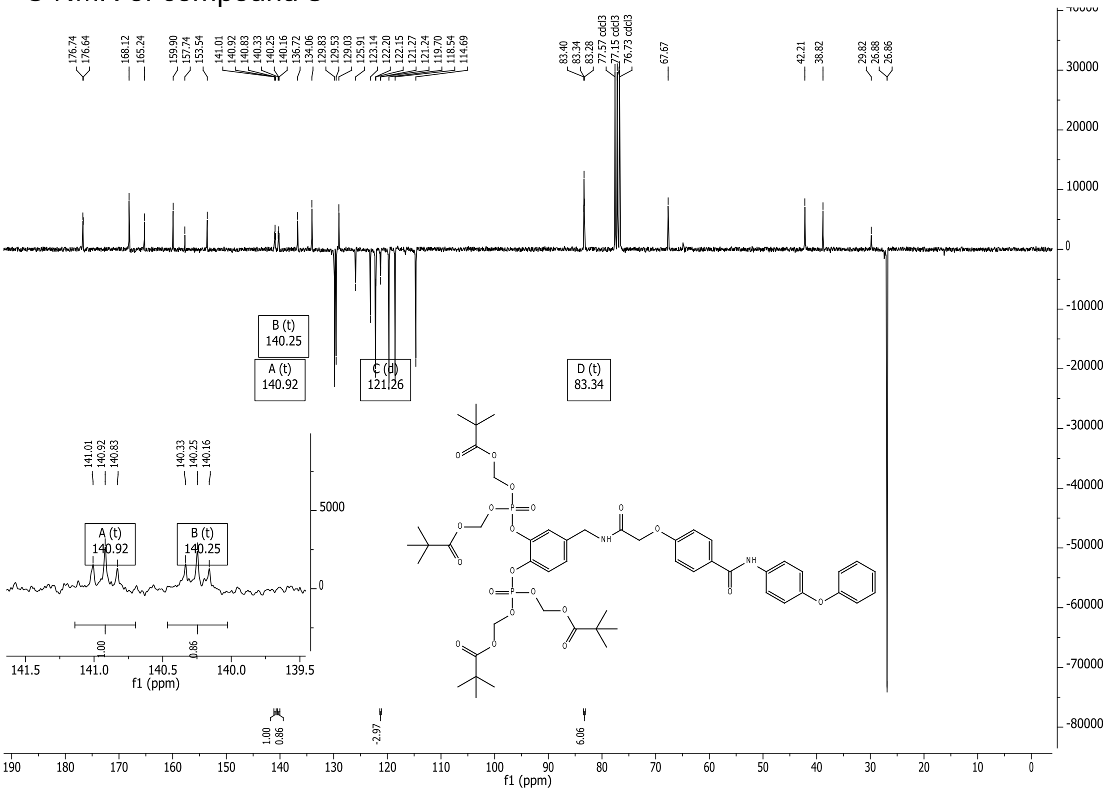

^{31}P NMR of compound **8**

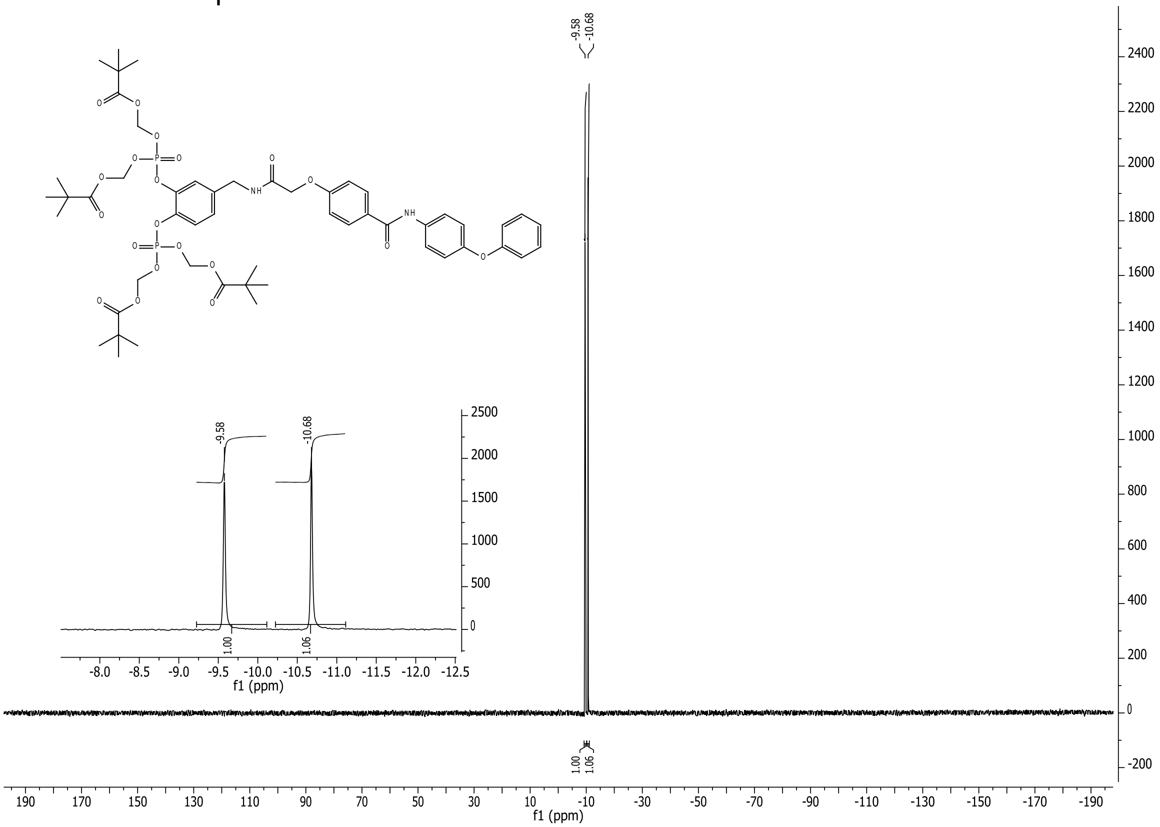

Supplementary References

1. Nikolovska-Coleska, Z. *et al.* Development and optimization of a binding assay for the XIAP BIR3 domain using fluorescence polarization. *Anal. Biochem.* **332**, 261-273 (2004).
2. Elumalai, N., Berg, A., Natarajan, K., Scharow, A. & Berg, T. Nanomolar Inhibitors of the Transcription Factor STAT5b with High Selectivity over STAT5a. *Angew. Chem. Int. Ed.* **54**, 4758-4763 (2015).
3. Elumalai, N., Berg, A., Rubner, S. & Berg, T. Phosphorylation of Capsaicinoid Derivatives Provides Highly Potent and Selective Inhibitors of the Transcription Factor STAT5b. *ACS Chem. Biol.* **10**, 2884-2890 (2015).
4. Gräber, M. *et al.* Oral Disinfectants Inhibit Protein-Protein Interactions Mediated by the Anti-Apoptotic Protein Bcl-xL and Induce Apoptosis in Human Oral Tumor Cells. *Angew. Chem. Int. Ed.* **52**, 4487-4491 (2013).
5. Reimers, F. Analytical chemistry of p-hydroxybenzoic acid esters. *Zeitschrift f. anal. Chemie* **122**, 404-418 (1941).
6. Feng, Z. W., Zhao, X. Q. & Bi, H. Selective esterification of non-conjugated carboxylic acids in the presence of conjugated or aromatic carboxylic acids over active carbon supported methanesulfonic acid. *Sci China Ser B* **51**, 990-992 (2008).
